# Characteristics of the Western Province, Zambia, trial site for evaluation of attractive targeted sugar baits for malaria vector control

**DOI:** 10.1186/s12936-024-04985-0

**Published:** 2024-05-18

**Authors:** Annie Arnzen, Joseph Wagman, Chama Chishya, Erica Orange, Thomas P. Eisele, Joshua Yukich, Ruth A. Ashton, Javan Chanda, Jimmy Sakala, Benjamin Chanda, Rayford Muyabe, Tresford Kaniki, Mwansa Mwenya, Gift Mwaanga, Will T. Eaton, Brooke Mancuso, Alice Mungo, Monicah M. Mburu, Nchimunya Bubala, Ackim Hagwamuna, Edgar Simulundu, Kochelani Saili, John M. Miller, Kafula Silumbe, Busiku Hamainza, Willy Ngulube, Hawela Moonga, Jacob Chirwa, Thomas R. Burkot, Laurence Slutsker, Megan Littrell

**Affiliations:** 1 PATH, Seattle, WA USA; 2 PATH, Washington, DC USA; 3 PATH, Kaoma, Zambia; 4 Centre for Applied Malaria Research and Evaluation, Tulane School of Public Health and Tropical Medicine, New Orleans, LA USA; 5 PATH, Lusaka, Zambia; 6 Present Address: Jhpeigo, Lusaka, Zambia; 7 Macha Research Trust, Choma, Zambia; 8 National Malaria Elimination Centre, Lusaka, Zambia; 9 Australian Institute of Tropical Health and Medicine, James Cook University, Cairns, QLD Australia; 10 Independent Consultant, Atlanta, GA USA

**Keywords:** Malaria, Vector control, Attractive targeted sugar bait, Site characterization, Residual transmission

## Abstract

**Background:**

The attractive targeted sugar bait (ATSB) is a novel malaria vector control tool designed to attract and kill mosquitoes using a sugar-based bait, laced with oral toxicant. Western Province, Zambia, was one of three countries selected for a series of phase III cluster randomized controlled trials of the Westham ATSB Sarabi version 1.2. The trial sites in Kenya, Mali, and Zambia were selected to represent a range of different ecologies and malaria transmission settings across sub-Saharan Africa. This case study describes the key characteristics of the ATSB Zambia trial site to allow for interpretation of the results relative to the Kenya and Mali sites.

**Methods:**

This study site characterization incorporates data from the trial baseline epidemiological and mosquito sugar feeding surveys conducted in 2021, as well as relevant literature on the study area.

**Results: Characterization of the trial site:**

The trial site in Zambia was comprised of 70 trial-designed clusters in Kaoma, Nkeyema, and Luampa districts. Population settlements in the trial site were dispersed across a large geographic area with sparsely populated villages. The overall population density in the 70 study clusters was 65.7 people per square kilometre with a total site population of 122,023 people living in a geographic area that covered 1858 square kilometres. However, the study clusters were distributed over a total area of approximately 11,728 square kilometres. The region was tropical with intense and seasonal malaria transmission. An abundance of trees and other plants in the trial site were potential sources of sugar meals for malaria vectors. Fourteen *Anopheles* species were endemic in the site and *Anopheles funestus* was the dominant vector, likely accounting for around 95% of all *Plasmodium falciparum* malaria infections. Despite high coverage of indoor residual spraying and insecticide-treated nets, the baseline malaria prevalence during the peak malaria transmission season was 50% among people ages six months and older.

**Conclusion:**

Malaria transmission remains high in Western Province, Zambia, despite coverage with vector control tools. New strategies are needed to address the drivers of malaria transmission in this region and other malaria-endemic areas in sub-Saharan Africa.

**Supplementary Information:**

The online version contains supplementary material available at 10.1186/s12936-024-04985-0.

## Background

Malaria transmission persists in many African countries despite high coverage with key malaria vector control tools, including insecticide-treated nets (ITNs) and indoor residual spraying (IRS) [1]. New vector control tools are needed to further reduce or eliminate transmission. The attractive targeted sugar bait (ATSB) is a promising new tool designed to complement ITNs and IRS for integrated malaria vector control. The ATSB is intended to attract and kill mosquitoes using a sugar-based bait laced with an oral toxicant [2, 3]. ATSBs have the potential to address drivers of malaria transmission as the bait station is accessible to outdoor sugar feeding mosquitoes. Furthermore, the oral toxicants in the version of ATSB tested in the trial employed a different mode of action than those exploited by ITNs and IRS [2, 4].

The Westham ATSB Sarabi version 1.2 was field tested through phase III cluster randomized controlled trials to evaluate the epidemiological impact in three malaria-endemic settings in Africa. These trials were conducted in Kenya (2022–2024), Mali (2022–2024), and Zambia (2021–2023) [2]. The trial sites were selected to represent a range of different malaria transmission settings relevant to malaria control programmes across sub-Saharan Africa. The trial site in the southern region of Ségou, Mali, is in western Africa and is characterized by a hot, Sahelian climate with intense and seasonal malaria transmission that lasts approximately six months. Villages in Mali are typically well-defined and densely populated. The trial site in Western Province, Zambia, is in southern Africa and is tropical with intense and seasonal malaria transmission that also lasts approximately six months. Population settlements in this rural area are geographically dispersed and often contain geographically large villages consisting of sparsely populated areas. The trial site in western Kenya is in east Africa and is temperate with perennial malaria transmission. This site has a higher population density as compared to Zambia but lower than villages within the Mali trial site. Each trial site has different dominant vector species as well as distinct local flora. These important variations across the trial sites are intended to produce evidence around ATSB efficacy in different contexts.

This case study describes key aspects of the environment, population, and malaria transmission context for the Zambia ATSB trial site. The Zambia site characterization summarizes important contextual information to facilitate interpretation and utilization of the trial results from Zambia and better understand the usefulness of this tool in other settings in sub-Saharan Africa.

## Methods

The characterization of the trial site in Western Province, Zambia, draws on a combination of primary and secondary data sources. A baseline epidemiological survey was conducted in 2021 across the trial site towards the end of the peak malaria transmission season (April–May 2021) and prior to the introduction of ATSBs. The survey measured parasite prevalence and key contextual variables including demographic information, economic profile, housing type, ITN and IRS coverage/use, and treatment-seeking behaviour for suspected malaria. The survey methods and results are described in detail elsewhere ([2], Ashton et al. pers.commun). The survey data were used in this paper to characterize household occupation, common housing type, treatment-seeking behaviour for suspected malaria, vector control intervention coverage, and malaria prevalence across the trial site. A 2021 field study was conducted in the vicinity of the trial site with a prototype attractive sugar bait (ASB) designed to attract malaria vectors but without the lethal insecticide. This field study was conducted to estimate ASB feeding rates among local malaria vectors. A detailed description of the feeding study methods and results are available elsewhere [3]. The data from the feeding study were used in this paper to describe the primary vectors, vector abundance, and feeding rates in the trial site.

Publicly available literature, project reports, and Government of Zambia reports were reviewed to characterize the trial site ecology, health system, COVID-19 situation, and malaria control strategies.

## Results: Characterization of the trial site

### Trial location

**Fig. 1:**
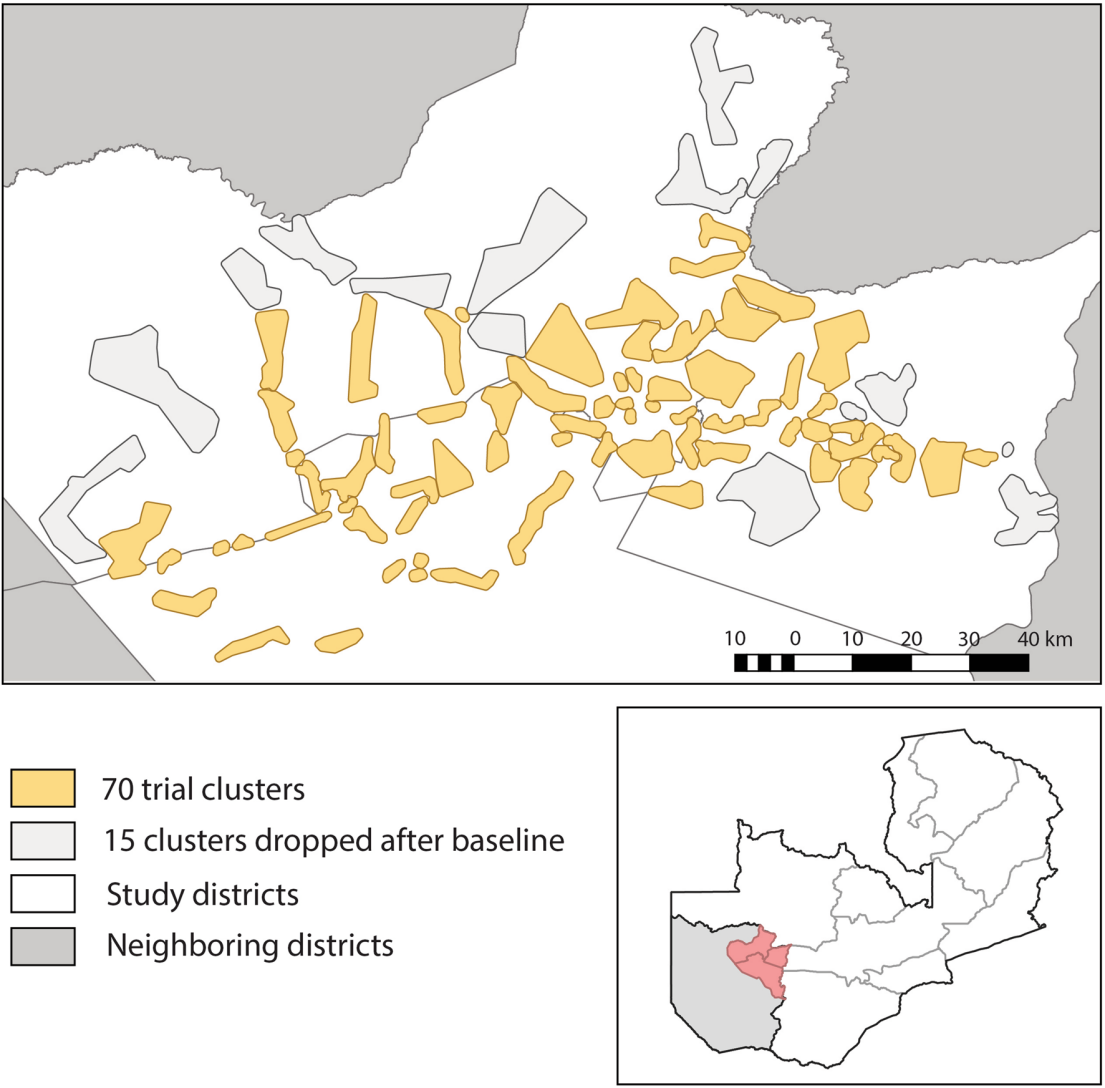
Map of the trial site located in Western Province, Zambia

The Zambia ATSB phase III trial site was located in three adjacent districts in Western Province: Kaoma, Luampa, and Nkeyema districts (Fig. 1). Western Province is approximately 400 kilometres from the capital of Zambia, Lusaka. Baseline data were collected in 85 rural clusters of which 70 were included in the trial. The 15 clusters included in the baseline and excluded from the main trial were dropped due to accessibility challenges, high refusals, or very low malaria prevalence. The 70 trial clusters cover a geographic area of approximately 1858 square kilometres, with a total bounded area of approximately 11,728 square kilometres. The clusters were created using a K means algorithm and satellite imagery to draw areas with a minimum of 250 households to meet sample size requirements (Ashton et al., pers.commun).

### Environment

#### Climate

Western Zambia has a tropical climate with an average annual rainfall of approximately 1000 mm; 93% of the total rainfall occurs between November and March [5, 6]. The total rainfall from November 2020 to March 2021 was 1139 mm (Kaoma Meteorological Station, unpublished data). The average daily maximum temperature during this period was 30.3 ºC and the average daily minimum was 18.9 ºC. A cool dry season typically occurs from May through August followed by a hot dry period from September through October. Total rainfall from May to October 2021 during the dry season was 59.6 mm, maximum daily temperature was 30.7 ºC, and minimum daily temperature was 11.6 ºC (Kaoma Meteorological Station, unpublished data).

#### Land cover and use

The dominant soils in Western Province are Kalahari sands which favour tree growth [6]. The study site districts of Kaoma, Nkeyema, and Luampa are predominantly Central Zambezian Miombo woodlands which are characterized by Miombo trees (*Brachystegia* genus). There were 339 different nectar producing plant species observed across the trial site (see Supplementary file 1 for the full information). In particular, the Miombo woodlands are rich in edible indigenous fruit trees [7]. These indigenous tree species are found in the larger landscape between households. Mango trees (*Mangifera indica*), which are planted for fruit and shade, are the most common household tree. The site also includes seasonally flooded grasslands and dry forests [6]. The two main water bodies in the trial site are the Luampa and Luena rivers. During the rainy season, flooding is common in areas surrounding the rivers, with pools forming as the flooding recedes.

Several tree and plant species found in the trial site are potential sources of sugar meals for malaria vectors. Trees appear to be the main source of sugar production. *Mangifera indica* (mango trees) and *Senna siamea* (evergreen trees) are predominant in the trial site and are known to be attractive to *Anopheles gambiae* [8]. Herbaceous plants that are known to be attractive to mosquitoes and are also common in the study site include *Bidens pilosa* (Blackjack), *Senna occidentalis* (coffeeweed)*, Manihot esculenta* (cassava) and *Ipomoea batatas* (sweet potato) (see Supplementary file 1 for the full information).

**Fig. 2:**
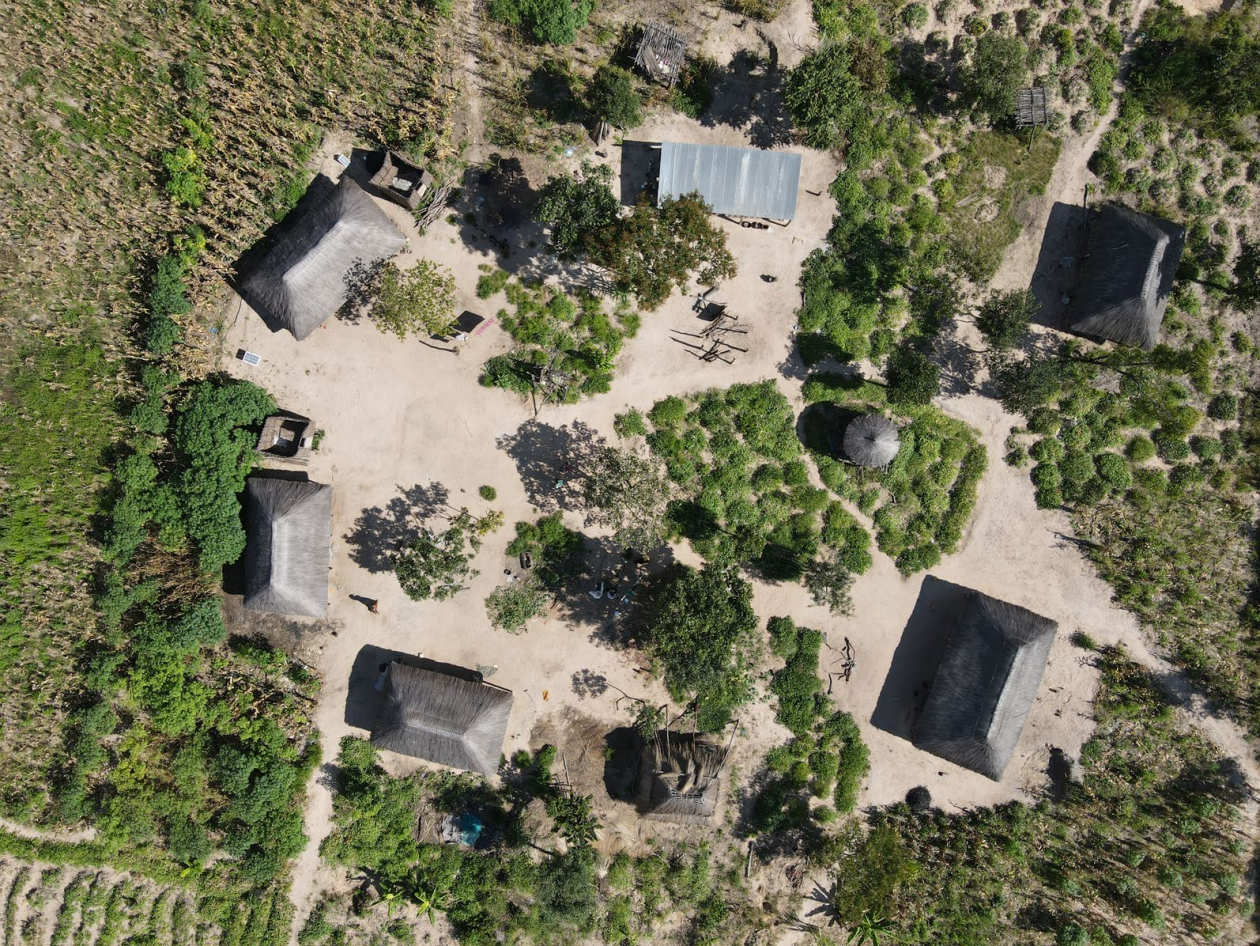
Drone image of vegetation surrounding household in the trial site

Households in this region rely primarily on commercial and subsistence farming. During the trial baseline study, 88% of households across the trial site reported farming or gardening as their primary occupation. The main crops grown were maize, cassava, rice, millet, and vegetables [9, 10]. A subset of the population also reared cattle. Other reported livelihoods include tobacco production in Nkeyema District and exploitation of trees for charcoal production. Households typically have agricultural plots near their living structure(s) to cultivate maize and cassava ranging in size from one to two hectares (see Supplementary file 2 for full report). Areas directly surrounding living structures are typically swept clean with little to no vegetation other than trees providing shade and/or fruit (see Fig. 2).

### Population

**Fig. 3:**
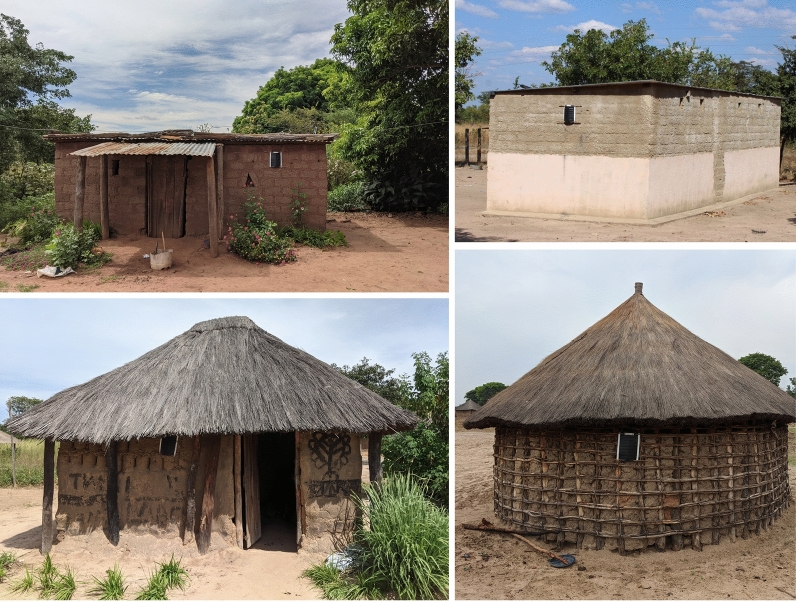
Commonly found house types in the trial site *Photo credit: Erica Orange*

The combined population of Kaoma, Nkeyema, and Luampa districts was estimated to be 246,785 during the 2022 national census [11]. A baseline enumeration of the study area estimated a population of 122,023 in the 70 trial clusters. Households were defined as a person or group of persons, related or unrelated, who live together in the same dwelling compound under one household head and share a common source of food. The average household size in Western Province was 4.7 people [11]. All trial clusters were rural. These clusters covered a range of geographic sizes and housing densities. In general, the trial clusters were sparsely built with approximately 0.25 structures per hectare (median 0.36 IQR (0.19–1.24) across clusters). The baseline trial survey found that houses were primarily constructed with thatch/leaf roofs (72%), walls with bamboo/wood and mud (46%), and earth/sand floors (86%). Some houses had corrugated iron roofs (28%) and walls made of stone with mud (30%) (see Fig. 3). For all house types, 21.1% had closed eaves and windows that are closed/sealed leaving many houses not fully sealed and with potential entry points for mosquitoes.

### Health system

There were three levels of health facilities available across the trial site including health posts (subdistrict level), health centres (district level), and hospitals (district level 1) (see table 1) [12]. There was a total of 29 health facilities across the trial site, of which the majority were health posts. Health posts are staffed by one or more community health assistants that typically have received a standardized 12 month training on disease screening and prevention [13]. During the baseline study, 97% of people within the study area reported seeking care for fever from a health facility inclusive of hospital, health centre, or health post.

### COVID-19

Zambia experienced four waves of COVID-19: July 2020, January to February 2021, June to July 2021, and December 2021 to January 2022 [14]. Following the fourth wave, Western Zambia recorded 18,887 cumulative cases and 129 deaths [15]. COVID-19 vaccines were introduced in Zambia in April 2021 with Johnson and Johnson and AstraZeneca being the predominant vaccines in the trial site Table 1.

**Table 1:** Overview of health facility type in the trial site

Facility type	Description
District hospital	• Serve a population of 80,000 to 200,000 people • District health officers provide services
Health center	• Serve a population of 10,000 people • Staffed by a clinical officer, nurse, or environmental health technician
Health post	• Serve a population of 3500 people in rural areas • Within 5 km radius for sparsely populated area • Staffed by community health assistants that also provide community-level services

Source: The National Community Health Strategy: 2019–2021 [13]

### Malaria

#### Malaria vectors

Overall, *Anopheles* spp. diversity in the study site was high. During pre-trial entomological feeding studies from March to May 2021 in a subset of ten clusters within Kaoma and Nkeyema districts, 14 different *Anopheles* species were morphologically identified from a total of 144,550 *Anopheles* mosquitoes collected in indoor and outdoor CDC ultraviolet light traps over 7,200 collection nights [3]. Of these, *An. funestus *sensu lato (*s.l*.), *Anopheles squamosus, Anopheles tchekedii*, and *Anopheles coustani* were the most abundant, with *An. gambiae s.l.* present in low numbers (Table 2).

**Table 2:** The mean number of *Anopheles* mosquitoes collected in US CDC ultraviolet light traps per household collection night, using aggregate totals from indoor and outdoor traps at the same household, from March to May, 2021 across the Zambia ATSB trial site

Species (Morphological ID)	Mean number of males	Mean number of females	Mean total number	% of total N = 20.08 %
*An. funestus s.l*	0.27	5.99	6.26	31
*An. squamosus*	0.02	4.02	4.03	20
*An. tchekedii*	0.01	3.80	3.81	19
*An. coustani*	0.01	3.76	3.77	19
*An. maculipalpis*	0.00	0.49	0.49	2
*An. gambiae s.l*	0.01	0.45	0.45	2
*An. brunnipes*	0.00	0.12	0.12	1
*An. rufipes*	0.00	0.11	0.11	1
*An. gibbinsi*	0.00	0.08	0.08	< 1
*An. pretoriensis*	0.00	0.01	0.01	< 1
*An. implexus*	0.00	0.00	0.00	< 1
*An. pharoensis*	0.00	0.00	0.00	< 1
Other & Not Identified^a^	0.01	0.72	0.72	4
Total	0.33	19.75	20.08	100

^a^Includes species with fewer than 10 specimens (*An. falvicosta*, *An. therileri*) as well as those in poor condition and not able to morphologically identify to species

During this study, three groups of mosquitoes tested positive for *Plasmodium falciparum* sporozoites assessed by standard *P. falciparum* enzyme-linked immunosorbent assay (ELISA) screening: *An. funestus s.l.* (sporozoite rate [SR] = 3.2%), *An. gambiae s.l.* (SR = 0.2%), and *An. squamosus* (SR = 0.2%) [3]. *Anopheles funestus s.l.* was both the most abundant *Anopheles* mosquito collected as well as the dominant malaria vector, representing 95% of all infectious mosquitoes collected *s.l.* in 2021. Subsequent molecular testing identified 100% of the positive *An. funestus* specimens as *An. funestus *sensu stricto (*s.s*.), and both positive *An. gambiae s.l.* specimens were identified as *Anopheles arabiensis*. Infectious *An. funestus s.s.* mosquitoes were equally likely to be collected in light traps set up indoors or outdoors [3].

An additional CDC ultraviolet light trap sampling effort conducted on separate nights collected mosquitoes that were screened for the presence of natural sugar meals using a cold anthrone test [16, 17]. The cold anthrone test identified mosquitoes that have recently (within several hours) acquired a natural sugar meal [16, 17]. Results suggested variable natural sugar feeding rates by species (Table 3). Though nearly half (47%) of all *An. funestus s.l.* screened were positive for a recent natural sugar meal, this proportion varied by cluster and by month, ranging from 19 to 67%.

**Table 3:** The proportion of mosquitoes positive for recent natural sugar meals from March to May, 2021, across the Zambia ASB trial site

Species	Number tested	Proportion positive %
*An. tchekedii*	1826	21
*An. funestus s.l*	1483	47
*An. coustani*	1283	13
*An. squamosus*	1037	10
*An. pharoensis*	673	5
*An. gibbinsi*	619	20
*An. brunnipes*	84	25
*An. pretoriensis*	61	28
*An. gambiae s.l*	30	23
*An. rufipes*	19	53
*An. implexus*	17	6
*Total*	7132	22

Finally, standard World Health Organization tube bioassay tests against alphacypermethrin, deltamethrin, and permethrin [18] indicated high levels of pyrethroid resistance in local *An. funestus s.l.* (24 h mortality ranging from 46 to 60%) and *An. gambiae s.l.* (24 h mortality ranging from 67 to 80%) from the study site. Insecticide resistance has not yet been detected for the primary chemical used for IRS in the trial area, clothianidin, nor for other IRS chemicals including pirimiphos-methyl. Resistance has also not been detected for the active ingredient used in the Sarabi 1.2 ATSB, dinotefuran [3].

#### Malaria burden

Malaria was endemic in the trial site and infections peaked following the annual rainy season [19]. In Western Province, the estimated malaria incidence was 785 per 1000 people in 2021 [19]. The trial baseline study found *P. falciparum* prevalence by rapid diagnostic test (SD Bioline Malaria Ag P.f, Standard Diagnostics, South Korea, and Abbott Bioline Malaria Ag P.f, Abbott, USA) was 50% among people ages six months and older during peak transmission season.

#### Other malaria vector control interventions in the trial site

**Fig. 4:**
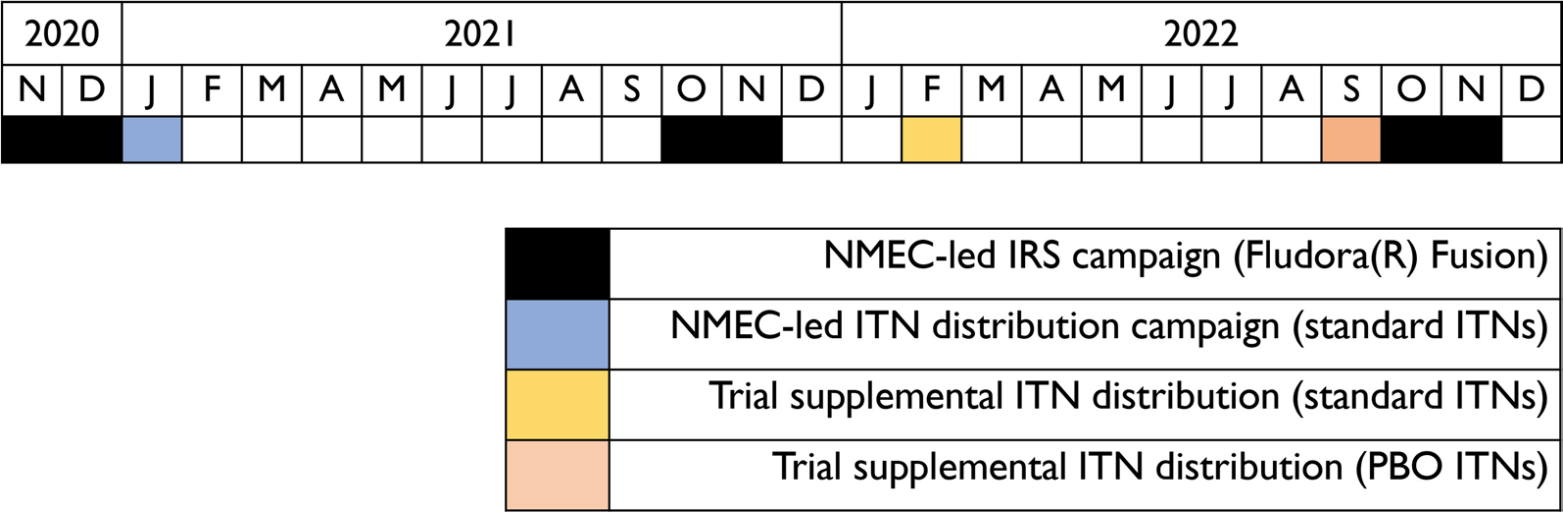
Overview of IRS and ITN campaigns in the trial site from 2020 through 2022

In the trial site, per the National Malaria Elimination Strategic Plan, universal access to vector control interventions was defined as households having access to one ITN per two people or IRS within the past 12 months [20]. Three IRS and ITN campaigns were conducted in the trial site using a mosaic approach between November 2020 and November 2022 (see Fig. 4). Before the start of the rainy season, the National Malaria Elimination Centre (NMEC) led annual IRS campaigns using Fludora^®^ Fusion WP-SB 56.25 (clothianidin and deltamethrin) in a subset of health facility catchment areas that were selected during an annual microplanning exercise. IRS was conducted in 42 of the clusters in 2020, 42 clusters in 2021, and 21 clusters in 2022. In 2020, the NMEC-led ITN campaign directly followed the IRS campaign and included distribution of PermaNet^®^ 2.0 (deltamethrin) ITNs. Two supplemental ITN distributions were implemented by the trial team within the study clusters. The first was in February 2022 and was implemented to address high demand for ITNs in the context of trial activities. A total of 28,908 PermaNet^®^ 2.0 (deltamethrin) ITNs were distributed as part of a strategy to distribute one ITN per household across the trial site. To address potential gaps in ITN coverage following the 2020 NMEC-led campaign, a total of 59,051 VEERALIN^®^LN (alpha cypermethrin plus pyrethroid piperonyl butoxide) ITNs were distributed in September 2022 to the 48 clusters that were not fully covered by NMEC-led IRS. During this distribution, one ITN was provided for every two residents in the household per the national strategy. As a result of these efforts, the trial site had high levels of coverage with vector control interventions with more than 60% of households having access to at least one ITN per two people or IRS.

#### Case management in the trial site

Across the trial site, about half (47%) of people of all ages with fever in the past two weeks reported seeking care from a formal provider during the baseline survey. Approximately 80% of people with a fever who sought treatment from a formal health provider reported being tested for malaria. In this setting, care for suspected malaria was sought almost exclusively from public/government health facility providers. A common limiting factor to the effectiveness of malaria case management is stockouts of key commodities including malaria rapid diagnostic tests and treatments. During the ATSB trial, the study team noted periods of commodity stockouts reported by study participants which may have contributed to suboptimal case management of suspected malaria across the trial site.

## Conclusion

The residents of the ATSB study clusters in Kaoma, Nkeyema, and Luampa districts of Western Province were predominantly subsistence farmers whose residences make them vulnerable to biting mosquitoes, with 14 morphological species present. High baseline prevalence despite high ITN and IRS coverage suggests a need for complementary vector control interventions against the primary malaria vector, *An. funestus s.l*. Evidence of recent natural sugar feeding was found in almost half of mosquitoes captured in CDC light trap collections with high variability in the prevalence of sugar by cluster and species, suggesting that ATSBs have the potential to have a significant impact, although impact may be highly variable depending on the availability of competing natural sugar sources.

The challenge of persistent, high levels of malaria transmission in the context of substantial ITN and IRS deployment is not unique to Zambia [21]. The reduction and ultimately elimination of malaria transmission in this context, and across many contexts in malaria-endemic sub-Saharan Africa, requires further investigation and investment in both existing and new tools and approaches. These include the ATSB product evaluated at the study site characterized in this paper as well as at the two additional study sites in Mali and Kenya where trials were conducted in 2022–2024. The site characterization presented here can guide interpretation of the results and application of learnings to other malaria-endemic areas in sub-Saharan Africa.

### Supplementary Information

Supplementary material 1.

Supplementary material 2.

## Data Availability

De-identified data are available from the corresponding author on reasonable request. Following publication of forthcoming secondary analyses of trial data, the deidentified trial dataset will be posted on a public repository.

## Abbreviations

ASB: Attractive sugar bait
ATSB: Attractive targeted sugar bait
ELISA: Enzyme-linked immunosorbent assay
IRS: Indoor residual spraying
ITN: Insecticide-treated net
NMEC: National Malaria Elimination Center
SR: Sporozoite rate
