# Supplementary material for: Characteristics of the Western Province, Zambia, trial site for evaluation of attractive targeted sugar baits for malaria vector control
Source: Malar J. 2024 May 18;23:153. doi: 10.1186/s12936-024-04985-0 (PMC11102358; doi:10.1186/s12936-024-04985-0)
Supplement: Supplementary file 1 — Supplementary material 1. [file 12936_2024_4985_MOESM1_ESM.pdf]

Rapid Assessment of Vegetation and Sugar Sources to Inform Cluster Creation

Kaoma, Luampa and Nkeyema Districts

Western Province, Zambia

PATH/Innovative Vector Control Consortium (IVCC)
Attractive Targeted Sugar Bait (ATSB) Project
June 2023

Nicholas Wightman

General Overview

The Innovative Vector Control Consortium (IVCC) is continuing the development of possible methods to reduce mosquito presence around habitation sites through the use of the Attractive Targeted Sugar Bait (ATSB) traps in Kaoma, Luampa and Nkeyema Districts of Western Province, Zambia. Between February and March 2021, a vegetation transect survey was conducted around 50 randomly selected households in Kaoma and Nkeyema Districts to ascertain the vegetation and species composition generally found around homesteads in the area as the flora around habitation sites provide natural sugar sources for feeding mosquitoes from local nectar producing plants.

The transect survey highlighted the range of indigenous and exotic trees that are commonly planted around the homesteads of the area as well as the annual agricultural activity around most households that selects for annual, or rarely perennial, plant species well suited to growing in sites with regular soil disturbance and compaction. The results were then analysed by project partners in the Department of Biology at Oxford University who were able to determine sugar availability per month and identify those times of year when competition from natural sugar sources would be at their highest with the ATSB traps.

Briefly, Chidumayo (1987) designates the flora area from the edge of Mumbwa district to Mongu as western drier Miombo, consisting of *Brachystegia spiciformis – Julbernardia paniculata* woodlands. Fanshawe (1969, 2010) further breaks down the flora as containing areas of dry evergreen forest (consisting of *Cryptosepalum exfoliatum* subsp. *pseudotaxus* and *Guibourtia coleosperma* dominants) and a mixture of Miombo and Kalahari woodland (dominated by *Brachystegia spiciformis, B. longiflora, Julbernardia paniculata, Erythrophleum africanum* and *Burkea africana*) together with areas of Chipya and Savanna woodlands where trees are spread at greater distances and tall grasses dominating the undergrowth. In addition, large areas of dambo grasslands border stream and river edges as well as low depressions flanking woodlands; these areas are dominated by grasses and sedges with perennial forbs and suffrutex species growing in lesser numbers. Termitaria are rare due to the high sand content of the soils and usually are only found in the miombo woodland areas where there is enough of a clay content to allow for the construction of their mounds.

In more recent times, the vegetation has changed with a rising population pressure exerted on the landscape, increasing clearance for agriculture (especially with increasing tobacco production in Nkeyema District) and continuing commercial exploitation of timber tree species and charcoal production. All these factors have, and are still changing the composition of woody vegetation of the Districts and its associated herbaceous flora.

The current work concentrated on the selection and surveying of possible study sites within the existing ATSB clusters for the identification of possible areas of high and low sugar availability in relation to sites of high and low building density. In total, sixteen study sites would be selected; four of high sugar and high building density; four of high sugar and low building density; four of low sugar and high building density; and four of low sugar and low building density.

Methodology

Using the shape files provided by PATH of current Clusters delineated for the Zambia ATSB trials occurring in Kaoma, Luampa and Nkeyema Districts, a desktop evaluation was carried out on suitable sites using Google Earth. Sites were selected based on the criteria of either high or low building density with the former quantified by containing a minimum of 30 households per unit area and the latter containing a minimum of 15 households per unit area. Following these criteria, 16 sites were identified for possible high building density (HBD) clusters and 20 sites were identified for possible low building density (LBD) clusters. Sites were selected from existing areas within current ATSB clusters created for the ATSB trial project as residents of these areas are already knowledgeable about the work that PATH has been conducting in the three districts.Proposed sites were named after the ATSB cluster that they were located in and given the acronym HBD or LBD depending on whether the site was a proposed high or low building density, respectively. In a few cases of the proposed LBD sites, more than one location was identified and these clusters were then given an additional numerical identification of 1 or 2 (i.e. Cluster 77 LBD 1 and Cluster 77 LBD 2).

Fieldwork was conducted between April 20th to 27th, 2023 and consisted of walking around each prospective site to observe and record the vegetation around and between the households and then to evaluate their occurrence within each site. The occurrence was recorded in one of four categories:

- Rare - three or fewer observations of each plant species across the site;
- Occasional - four to ten observations of each plant species across the site;
- Common - more than ten observations of each plant species across the site;
- Widespread - a plant species observed to occur in large populations (usually existing as a dominant part of the habitat) across the site.

Of the 36 proposed sites identified during the desktop evaluation, 35 sites were examined during fieldwork. At each site, the author was accompanied by a PATH Kaoma team member and a Community Health Worker from the cluster that was being surveyed. One site was not examined (Cluster 55 LBD 1) due to the low uptake of community participation in the ATSB project as well as a high abandonment rate of homesteads observed at site; thus it was decided that due to both issues, the site would not be surveyed if there was a high chance that the community would reject participation in the proposed project in addition to the possibility that the number of households may not meet the minimum number required by the low building density criterium.

Pictures taken during fieldwork activities were uploaded to the author’s Google Drive and shared with partners from PATH and Oxford team to help give better visualisation of each site. Data from each site was subsequently entered into a spreadsheet recording the species, family, habit (annual, tree etc.), status (indigenous or exotic), occurrence (rare, occasional etc.) and any further notes on the plant. A list of all species recorded across the HBD or LBD sites was also created with known flowering times of each species to help inform times of possible natural sugar availability.

Results and Discussion

Vegetation

Across all sites, a total of 339 different nectar producing plant species were observed from approximately 80 plant families. Of the total number of species, 108 are exotic in origin and 231 are indigenous with a breakdown of 89 herbaceous annual species, 97 herbaceous perennial species and 153 woody shrub or tree species. The highest species count from a single site numbered 115 plant species while the lowest species count numbered 51 plant species with an overall average of 85 plant species per site.

However, species composition per site is affected most by the age of the settlement. Older settlements are characterised by large mature exotic fruit trees, almost exclusively Mango (*Mangifera indica*), located around each homestead with the spaces between trees populated by annual grasses and weedy species tolerant of shade, while any sunny areas are usually sown to some small plots of crop plants (Figure 1). Occasionally a household will have also planted some well known exotic ornamental tree, shrub or perennial within the confines of the property boundary but this is usually in the minority across the settlement (Figure 2). Homesteads within older settlements are usually closely bordered by neighbouring homesteads of an almost identical vegetative makeup leading to a rather predictable floral assemblage of exotic fruit trees, crops and annual weedy herbs.

By contrast, younger settlements are composed of a more diverse mosaic of indigenous species in addition to the usually exotic and weedy annual species. With the establishment of a new homestead, residents will usually plant one to several exotic fruit trees, which will usually take between 10 to 20 to grow to a size that will provide shade in addition to fruit. However, while those young fruit trees are growing, several decently sized existing indigenous trees are usually left uncut to provide areas of shade for the household in the interim. Similarly, the land surrounding the homestead may be cleared to facilitate agricultural activities but root systems of existing woody species and even some herbaceous perennial species can persist, resprouting each year and growing alongside the crop plants. As a result, the vegetation around younger settlements is more varied.

Ultimately, the vegetation around households in the observed sites is impacted by human activities to some degree. The diversity of the indigenous woody component is gradually reduced with species selected for their ability to provide shade or fruits, with *Cryptoepalum exfoliatum* subsp. *pseudotaxus, Julbernardia paniculata* and *Terminalia brachystemma* subsp. *brachystemma* regularly providing the former and *Diospyros batocana, Strychnos pungens* and *Vangueriopsis lanciflora* regularly providing the latter. However, households usually also plant exotic fruits, with the most common being mango (*Mangifera indica*) but also regularly observed were guava (*Pidium guajava*), Citrus (*Citrus* cvs.), White Sapote (*Casimiroa edulis*) and Mulberry (*Morus alba*). With the growth of the exotic fruit trees, they eventually compete for space and cast greater shade providing both fruit and shade to the household and becoming more valuable than the indigenous trees; thus, gradually the exotic fruit trees predominate around older settlements and the indigenous woody species are replaced.

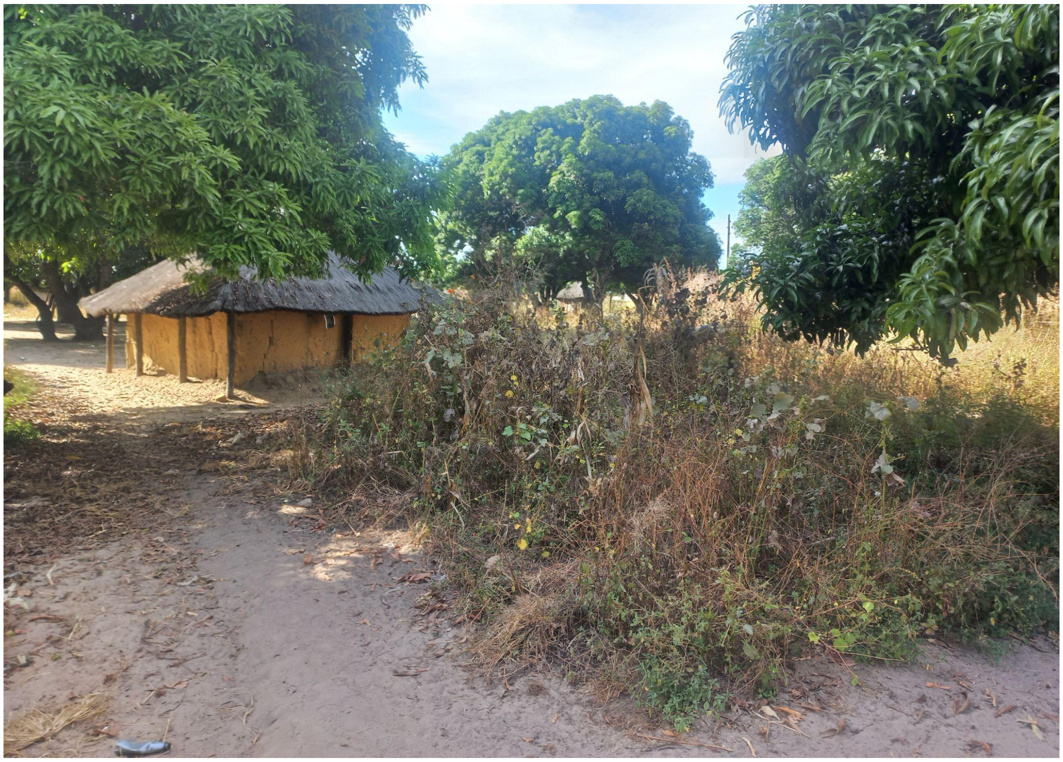

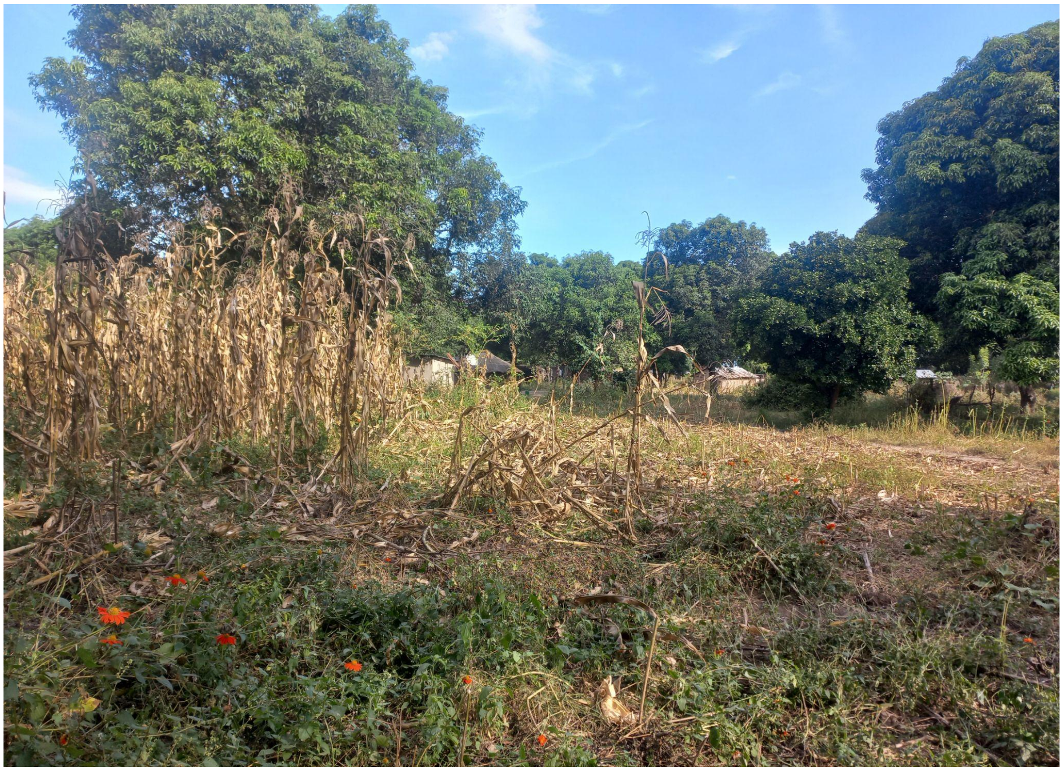

Figure 1. Older settlements at Cluster 39 HBD (top) and Cluster 85 HBD (bottom) characterised by large trees surrounding the homesteads and open spaces either overgrown by shade tolerant annual grasses and weeds (top) or sown with small patches of crop plants (like here with maize) in areas with adequate sunlight (bottom) [Photographed by N. Wightman, April 2023].

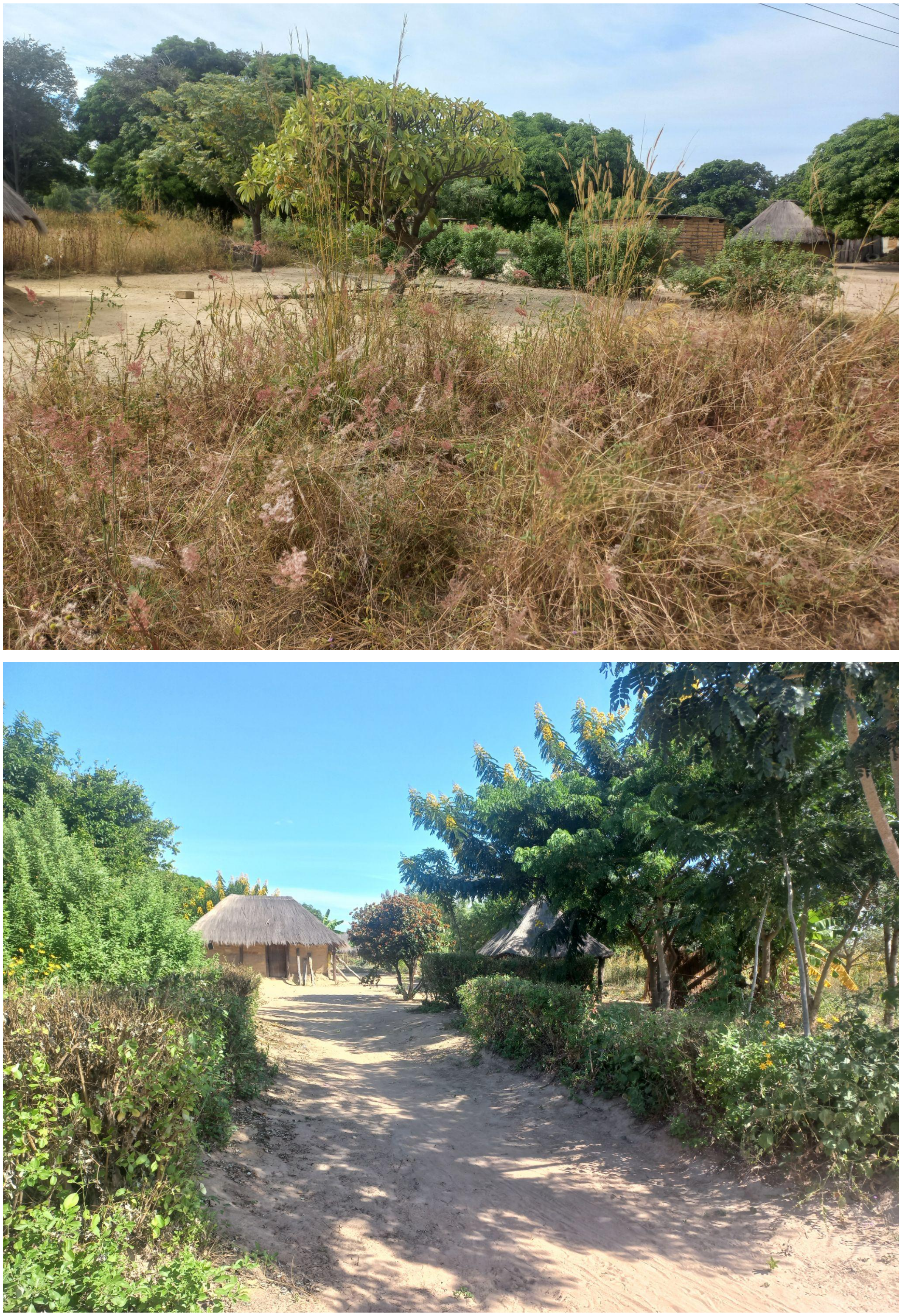

Figure 2. Homestead in which the owners have planted Frangipani (Plumeria cvs.), Senna siamea and maintain a hedge of Vitex negundo between them and a heighbouring homestead (top); a homestead with an avenue of ornamentals leading to the house including a hedge of Vitex negundo, several specimens of Senna spectabilis, Albizia lebbeck and Bixa orelleana (bottom) [Photographed by N. Wightman, April 2023].

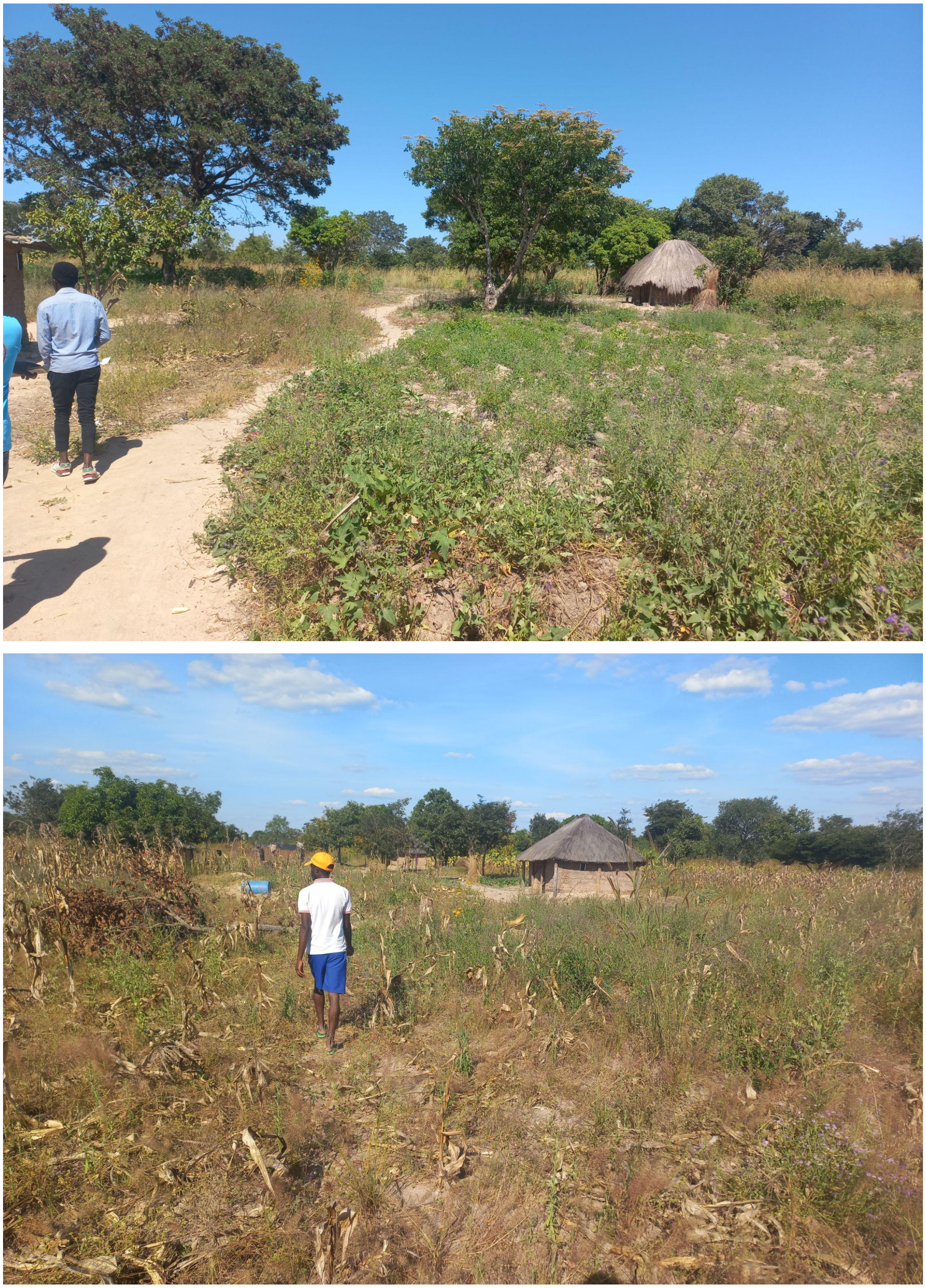

Figure 3. Homestead in Cluster 77 HBD with sweet potato growing adjacent to the hut and *Julbernardia paniculata* and *Terminalia mollis* acting as shade trees as young mango trees (*Mangifera indica*) grow just behind the hut to the right (top); and a homestead in Cluster 56 LBD 1 with maize field adjacent to the house and specimens of *Strychnos pungens, Bobgunnia madagascariensis, Burkea africana* left growing near the household for shade (bottom) [Photographed by N. Wightman, April 2023].

On the other hand, tilling the land for agriculture disturbs the natural soil conditions and reduces the ability for many of the herbaceous woodland annuals and perennials to sustain growth and regeneration but coppice regrowth of the woody species from established root systems will continue to occur. With the disturbance of the soil during agricultural activities, exotic and indigenous annual plant species adapted to such disturbed soils will start to dominate. Exotic annual species including *Acmella radicans, Amaranthus* spp., *Bidens pilosa, Galinsoga parviflora, Nicandra physalodes, Richardia scabra, Senna occidentalis, Tithonia rotundifolia* and *Xanthium strumarium* as well as the indigenous annual species *Bidens schimperi, Cleome hirta, Crotalaria goreensis, Indigofera astragalina, Indigofera nummularifolia, Vernonia meiostephana* and *Vernonia petersii* were all regularly encountered around households across most sites during fieldwork.

By comparison, few herbaceous perennials were regularly observed around households across sites. The exotic weeds *Boerhavia diffusa, Desmodium uncinatum, Tithonia diversifolia* and *Tridax procumbens* as well as the indigenous weeds *Achryanthes aspera, Pavonia urens* and *Sida alba* were commonly encountered with the last species, Sida alba, usually maintained around the household as the stems are bunched together and used as a makeshift broom. However, most perennials encountered around households are food plants like Banana (*Musa* cvs.), sweet potato (*Ipomoea batatas*) and Cassava (*Manihot esculenta*) or exotic ornaments like *Catharanthus roseus*, *Coreopsis lanceolata*, a semi succulent *Plectranthus* species and *Eleutherine bulbosa*. On occasion, shrubby weed species such as *Tithonia diversifolia* and *Lantana camara* were also observed growing as a hedge.

<u>Cluster Evaluation</u>

The creation of the four categories of high and low building density against high and low sugar availability has been discussed widely between the author and certain members of the PATH and Oxford teams. From the 2021 household transect survey work in Kaoma and Nkeyema Districts, the Oxford team were able to highlight a number of plant species identified as possessing high levels of natural sugar in addition to determining the months of the year in which natural sugar levels would be at their greatest (Figure 4). The flowering time of the different plant types explains the peaks in monthly sugar availability. In Zambia, many of the annual plant species will have their main flowering cycle starting around the period of February to March as the rains are tapering off; while, many the tree species will start their flowering cycle between July to October, a strategy that usually results in their fruits and seeds ripening as the new rainy season begins.

Many of the tree and annual species mentioned in slide 10 of Figure 4, as highlighted in the above Vegetation section, were commonly encountered across the potential sites during fieldwork. Table 1 and Table 2 document the occurrence of these species within the potential HBD and LBD sites, respectively. Of the thirty one species listed in Tables 1 and 2, nine are full sized trees, one is a suffrutex shrub, three are herbaceous perennial and eighteen are herbaceous annuals; in addition, twenty of the species are indigenous while 11 are exotic. The evaluation of sites as high or low sugar availability is then predominantly assessed mainly on these highlighted species and their occurrence within each site; furthermore, a greater emphasis is placed on those species more commonly found within the direct vicinity of households as observed during fieldwork.

Results: Sugar availability in Zambia per month

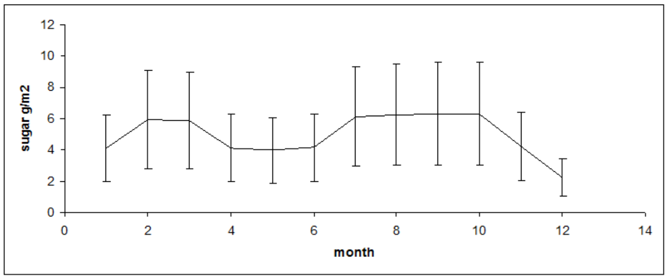

Zambia – 2020 Monthly mean landscape nectar sugar (g/m^2) and standard deviation

Key findings:

- The resulting maps from Zambia indicate clear heterogeneity in nectar resource across both landscapes in space and time.
- Trees appear to be responsible the main production of sugar/m^2 in Zambia. Our modelling indicates that tree flowers provide up to 100x more sugar resource per unit area than the local herbaceous vegetation.
- Trees that are predominant in the study sites and known to be attractive from the systematic evidence review to *An. gambiae* include: *Mangifera indica* and *Senna siamea.*
- Herbaceous plants that are predominant in the study sites and known to be attractive from the systematic review include: *Bidens pilosa*, *Senna occidentalis, Manihot sculenta* and *Ipomoea batatas* in Zambia.

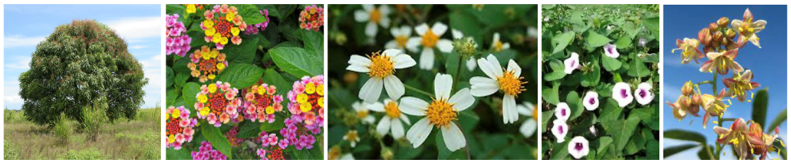

Next steps for new project

1. To identify clusters in the study sites with high and low levels of sugar nectar.
2. Ideally, we are looking for areas with a contrast in vegetation (more and less abundant). Since trees seem to be responsible of the main production of nectar, high levels of sugar sites should include more trees than low levels of sugar sites.
3. Herbaceous species with high levels of sugar:
   - Asteraceae: *Acanthospermum glabratum, Acanthospermum hispidulum, Laggera crispata*, Bidens pilosa, Bidens schimperi, Vernonia meiostephana, Vernonia perrottetii***, *Acmella radicans, Ageratum conzyoides, Emilia abyssinica, Gutenbergia gossweileri, Tithonia rotundifolia, Tridax procumbens.*
   - Fabaceae: *Indigofera astragalina, Indigofera nummulariifolia, Zornia glochidiata*.*
   - Other species: *Sida alba* (Malvaceae); *Celosia brevispicata, Celosia trigyna* (Amaranthaceae); *Biophytum umbraculum* (Oxalidaceae); *Boerhavia coccinea* (Nyctaginaceae); *Clerodendrum buchneri* (Lamiaceae).
4. Trees species with high levels of sugar:
   Mangifera indica (Anacardiaceae); *Brachystegia longifolia, Cryptosepalum exfoliatum, Julbernardia paniculate, Pterocarpus angolensis,* (Fabaceae); *Parinari curatellifolia* (Chrysobalanaceae); *Terminalia brachystemma, Terminalia mantaly* (Combretaceae).

*Highlighted in green: species of plants identified as potential sources for mosquitoes in Nick's report.
**Underlined: species of plants with the highest levels of sugar.

Figure 4. Select slides from the presentation 'Developing a new methodology to estimate the spatial distribution of sugar resource a study region in Zambia' with slide 7 highlighting natural sugar availability per month (top); slide 9 highlighting key findings (middle); and slide 10 highlighting plant species identified as containing high levels of natural sugar (bottom) [Source: Eva Herreros Moya, University of Oxford, March 2023].

One of the key findings in Slide 9 of Figure 4 state that 'trees appear to be responsible for the main production of sugar/m^2' and that 'modelling indicates that tree flowers provide up to 100x more sugar resource per unit area than the local herbaceous vegetation'. Of the nine identified tree species, six are indigenous and form a regular component of the natural Miombo and/or Kalahari woodland vegetation that occur within the three study districts; these species are *Brachystegia longifolia, Cryptosepalum exfoliatum* subsp. *pseudotaxus, Julbernardia paniculata, Parinari curatellifolia, Pterocarpus angolensis* and *Terminalia brachystemma* subsp. *brachystemma*. These indigenous species are not intentionally planted but rather naturally regenerating and were observed across sites as coppice regrowth through to mature trees; as a result, their recorded occurrences in Tables 1 and 2 reflect observations near households and in the greater landscape away from household vicinities. The remaining three tree species are exotic in origin and include *Mangifera indica, Senna spectabilis* and *Terminalia mantaly*. The three exotic species are intentionally planted by households to provide fruit, shade and ornamentation and were exclusively found within the vicinity of households and were not observed to have naturalised into the greater landscape. Therefore, the recorded occurrence of these exotic tree species strictly relates to observations within the vicinity of households.

Herbaceous vegetation, though stated above to provide less sugar/m^2 compared to trees, are nonetheless the most numerous both in the number of species observed but also the volume of individual plants observed, especially in the case of the herbaceous annual species. The vegetation transect survey conducted around households in Kaoma and Nkeyema Districts in 2021 by the author for PATH observed that many of these annual species ranged from a few specimens/m^2 to hundreds/m^2 in the most extreme cases. The adaptation to disturbed soils favours the growth and proliferation of many of the annual species listed in Tables 1 and 2, both around households as well as in disturbed areas of the greater landscape. There were a number of annual species regularly observed to occur widespread across most sites in both HBD and LBD areas including *Acanthospermum hispidum, Bidens pilosa, Bidens schimperi, Indigofera astragalina, Indigofera nummularifolia* and *Vernonia meiostephana,* which, with the exception of the last species have developed epizoochory seed dispersal mechanisms and spread through contact with humans. Moreover, special attention should be drawn to the species *Sida alba* as, though it is a weed of disturbed ground, it is regularly maintained around households for the use of its stems, which are cut, bundled and used as makeshift brooms to sweep around the household. In the final analysis, herbaceous species cannot compete with woody species on the amounts of flowers produced per season in addition to the regularity of producing those flowers as herbaceous plants are more subject to cultural control and removal when they don't provide a direct benefit to a household.

The discussion on the selection of sites that meet both the high and low sugar availability criteria have been ongoing since discussions with the author first started regarding the current project. Sites meeting the high sugar criteria were never in doubt due to the treed nature of many households in the districts but concern was expressed that meeting the criteria for low sugar would be the main challenge. Through consultation, it was concluded that seeking sites that would be considered heavily treed for the high sugar criteria and lightly treed for the low sugar criteria.

Table 1. High sugar producing plant species occurrence for the proposed high building density cluster sites. Occurrence initials as follows: W - Widespread; C - Common; O - Occasional; R - Rare.

**Plant species**	**Potential HBD Cluster Sites**															
	**24**	**29**	**33**	**39**	**41**	**45**	**55**	**56**	**64**	**69**	**73**	**77**	**80**	**83**	**84**	**85**
Mangifera indica (Anacardiaceae)	O	W	C	W	C	W	C	C	C	C	O	C	C	C	C	W
Brachystegia longifolia (Fabaceae - Caesalpinioideae)	O	-	-	O	O	-	C	-	-	C	C	O	C	O	C	C
Cryptosepalum exfoliatum subsp. *pseudotaxus* (Fabaceae - Caesalpinioideae)	O	O	C	O	O	R	C	O	O	-	C	O	-	C	C	-
Julbernardia paniculata (Fabaceae - Caesalpinioideae)	O	-	C	O	C	C	C	C	C	C	C	C	C	O	C	O
Pterocarpus angolensis (Fabaceae - Papilionoideae)	-	O	O	O	-	-	C	-	C	-	-	O	O	O	O	-
Parinari curatellifolia (Chrysobalanaceae)	C	-	O	C	-	O	-	C	C	C	C	O	-	-	O	O
Senna spectabilis (Fabaceae - Caesalpinioideae)	-	O	-	R	O	O	C	C	O	O	O	C	C	-	-	-
Terminalia brachystemma subsp. brachystemma (Combretaceae)	O	O	O	-	O	-	C	-	O	-	O	C	R	O	-	-
Terminalia mantaly (Combretaceae)	R	-	R	R	-	R	R	-	R	-	O	O	R	-	-	R
Acanthospermum glabratum (Asteraceae)	W	C	-	-	W	W	W	C	-	-	-	-	C	C	C	-
Acanthospermum hispidum (Asteraceae)	C	W	W	W	W	W	W	W	C	C	-	W	C	W	C	C
Acmella radicans (Asteraceae)	-	-	-	-	O	O	-	-	C	C	C	C	W	-	-	W
Ageratum conyzoides (Asteraceae)	-	-	-	-	-	-	O	-	-	-	O	O	C	-	-	O
Bidens pilosa (Asteraceae)	-	W	W	W	W	W	W	C	O	O	C	W	W	W	W	W
Bidens schimperi (Asteraceae)	-	O	W	W	W	W	W	W	C	C	C	W	-	W	C	C
Biophytum umbraculum (Oxalidaceae)	-	-	-	-	-	-	-	C	O	-	-	-	-	-	-	-

Boerhavia coccinea (Nyctaginaceae)	-	-	O	-	O	O	C	-	C	-	O	C	-	O	-	C
Celosia brevispicata (Amaranthaceae)	-	O	O	C	-	-	-	-	O	-	-	-	-	-	-	-
Celosia trigyna (Amaranthaceae)	-	-	-	-	-	-	-	-	-	-	W	O	W	-	-	-
Clerodendrum buchneri (Lamiaceae)	-	-	-	-	C	O	O	C	C	O	O	R	-	O	-	-
Gutenbergia gossweileri (Asteraceae)	-	-	-	O	-	-	O	C	-	-	-	O	-	-	-	-
Indigofera astragalina (Fabaceae - Papilionoideae)	C	W	W	W	W	W	C	W	C	W	W	W	C	W	W	W
Indigofera nummularifolia (Fabaceae - Papilionoideae)	C	W	C	W	W	W	W	W	C	C	O	C	-	W	W	C
Laggera crispata (Asteraceae)	-	-	C	C	W	W	W	C	C	-	-	C	W	-	-	C
Senna occidentalis (Fabaceae - Caesalpinioideae)	C	-	-	-	C	O	-	O	O	O	-	-	O	-	C	C
Sida alba (Malvaceae - Malvoideae)	-	C	C	C	W	W	C	C	C	C	C	C	C	-	C	C
Tithonia rotundifolia (Asteraceae)	-	W	W	W	W	W	-	O	O	O	-	-	-	-	-	W
Tridax procumbens (Asteraceae)	-	-	O	-	-	-	-	-	-	-	-	O	W	-	-	C
Vernonia meiostephana (Asteraceae)	-	-	W	W	W	W	W	W	W	C	W	W	-	-	W	W
Vernonia perrottetii (Asteraceae)	-	-	W	C	-	C	C	C	-	O	C	C	-	-	-	-
Zornia glochidiata (Asteraceae)	-	C	C	C	W	W	W	-	C	C	C	C	O	C	-	C

Table 2. High sugar producing plant species occurrence for the proposed low building density cluster sites. Occurrence initials as follows: W - Widespread; C - Common; O - Occasional; R - Rare.

Plant species	Potential LBD Cluster Sites																		
	**23**	**28**	**33**	**36**	**38 1**	**38 2**	**40 1**	**40 2**	**41**	**42**	**55**	**56 1**	**56 2**	**64**	**69**	**73**	**77 1**	**77 2**	**85**
Mangifera indica (Anacardiaceae)	C	C	C	C	C	C	O	O	C	C	C	C	R	C	O	C	C	C	O
Brachystegia longifolia (Fabaceae - Caesalpinioideae)	R	-	R	O	O	O	O	O	C	O	C	O	C	O	C	C	O	O	-
Cryptosepalum exfoliatum subsp. *pseudotaxus* (Fabaceae - Caesalpinioideae)	O	O	W	O	O	-	C	C	-	O	C	O	C	O	O	-	-	-	-
Julbernardia paniculata (Fabaceae - Caesalpinioideae)	O	C	W	C	C	C	C	-	C	C	C	C	C	C	C	C	O	C	O
Parinari curatellifolia (Chrysobalanaceae)	O	O	O	O	O	O	-	O	C	O	C	C	C	C	C	O	O	C	O
Senna spectabilis (Fabaceae - Caesalpinioideae)	-	-	-	-	-	-	-	-	R	O	O	O	R	-	O	O	-	O	-
Terminalia brachystemma subsp. brachystemma (Combretaceae)	C	C	-	O	O	-	C	-	O	-	-	O	O	O	O	-	-	-	-
Terminalia mantaly (Combretaceae)	-	-	-	R	-	-	-	-	R	-	-	-	-	O	-	-	R	-	R
Acanthospermum glabratum (Asteraceae)	-	-	-	-	C	W	C	-	W	W	-	-	-	-	-	C	-	-	C
Acanthospermum hispidum (Asteraceae)	W	W	W	C	W	W	C	C	W	W	C	C	C	C	C	W	C	-	C
Acmella radicans (Asteraceae)	C	-	-	C	-	-	-	O	C	C	O	O	-	W	C	C	C	-	-
Ageratum conyzoides (Asteraceae)	-	-	-	-	-	-	-	-	O	-	C	-	-	-	-	-	-	-	-
Bidens pilosa (Asteraceae)	C	W	C	C	W	-	-	C	W	W	C	C	C	W	C	C	C	C	W
Bidens schimperi (Asteraceae)	W	-	C	W	C	-	W	W	W	W	C	C	W	C	O	O	W	C	C
Biophytum umbraculum (Oxalidaceae)	-	-	-	-	O	-	C	-	-	C	C	-	C	-	-	O	-	-	O
Boerhavia coccinea (Nyctaginaceae)	O	-	-	C	C	-	-	-	C	O	-	-	C	C	C	-	O	O	C

Celosia brevispicata (Amaranthaceae)	-	-	-	-	O	O	O	-	-	C	-	-	-	-	-	-	-	-	O
Celosia trigyna (Amaranthaceae)	O	-	-	-	R	-	-	-	-	-	-	-	-	O	O	-	-	O	-
Clerodendrum buchneri (Lamiaceae)	-	-	-	O	O	O	O	-	-	C	-	-	-	C	-	O	O	-	-
Gutenbergia gossweileri (Asteraceae)	O	-	O	-	-	O	-	-	-	O	O	-	C	O	-	-	O	-	-
Indigofera astragalina (Fabaceae - Papilionoideae)	W	W	C	O	W	C	O	C	O	W	C	C	C	W	C	W	W	W	C
Indigofera nummularifolia (Fabaceae - Papilionoideae)	W	W	C	W	W	C	C	W	W	C	C	C	O	C	C	C	C	C	W
Laggera crispata (Asteraceae)	-	-	C	W	C	C	W	-	C	C	C	C	C	C	C	C	C	C	C
Senna occidentalis (Fabaceae - Caesalpinioideae)	-	O	O	O	-	-	O	-	-	O	-	-	-	-	-	-	-	O	-
Sida alba (Malvaceae - Malvoideae)	-	C	C	C	W	C	-	C	C	C	O	O	O	C	O	O	C	C	C
Tithonia rotundifolia (Asteraceae)	O	-	-	W	-	-	-	-	-	O	-	-	-	O	C	-	-	-	C
Tridax procumbens (Asteraceae)	-	-	-	-	O	-	-	-	O	-	O	-	-	-	C	-	O	O	C
Vernonia meiostephana (Asteraceae)	W	W	C	W	W	-	C	W	W	W	C	C	C	W	C	C	W	C	-
Vernonia perrottetii (Asteraceae)	O	O	C	-	-	O	O	C	-	O	C	-	O	O	O	-	C	O	-
Zornia glochidiata (Asteraceae)	-	-	C	C	W	C	C	C	W	C	C	-	C	C	-	-0	C	C	C

Final Cluster Selection

The desktop survey identified sixteen possible high building density areas and twenty possible low building density areas across the three study districts based on the clusters previously created for the initial ATSB trials. According to the Oxford University team's assessment of species, tabulated from the results of the 2021 household vegetation transect surveys, sugar availability is particularly affected by the presence of trees surrounding the households. Tables 1 and 2 list the same high sugar producing species as determined by the Oxford University team based on the 2021 transect work. As stated above, the indigenous tree species, though regularly observed across the different sites, were not regularly observed as household trees but rather occur in the larger landscape between households. Exotic tree species, on the other hand, were only observed as household trees with mango, *Mangifera indica,* by far the most common household tree. Mango trees are regularly planted for its fruit and then valued for the shade it provides to households as it grows larger, often supplanting other indigenous and exotic household shade trees.

Selection of the final clusters based on building density and sugar availability was a combined effort based on the desktop survey and subsequent ground truthing fieldwork surveys in addition to the analysis of species sugar indexes, flowering and fruiting phenology and rank abundance evaluations. The data collected by the author during fieldwork was provided to Eva Herreros Moya of the Oxford University team for the overall analysis of each cluster site to determine cluster suitability based on sugar availability versus building density. Table 3 lists the final cluster selection for sites in the study area according to building density and sugar availability and Figure 5 gives a visual representation of the clusters across the study area. The final selected clusters were made using the best data available based on fieldwork and desktop assessments.

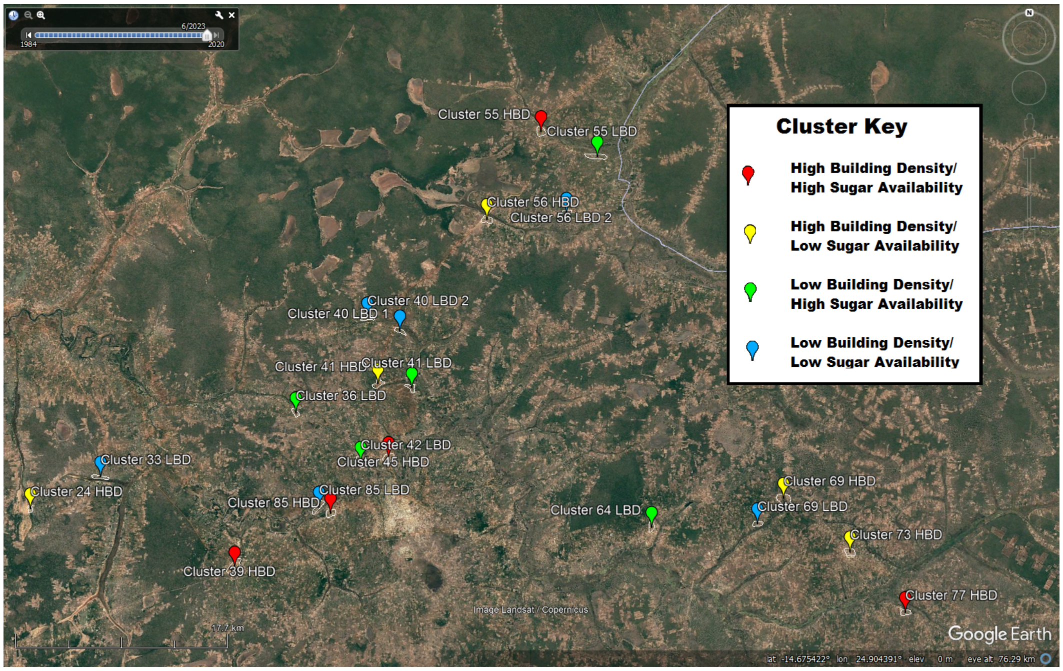

Figure 5. Visual representation of the different cluster locations across the study area of Kaoma, Luampa and Nkeyema Districts of Western Province, Zambia (Image: Google Earth, June 2023).

Table 3. Final cluster selection of sites for proposed future mosquito feeding study contrasting high and low rates of building density versus high and low rates of sugar availability.

Cluster	GPS coordinates	Building Density	Sugar Availability
39	-14.851911,24.677261	High	High
45	-14.76847,24.795538	High	High
55	-14.523339,24.910202	High	High
77	-14.879139,25.197626	High	High
85	-14.810619,24.751114	High	High
24	-14.81024,24.517987	High	Low
41	-14.713607,24.786573	High	Low
56	-14.589495,24.869226	High	Low
69	-14.794924,25.1018	High	Low
73	-14.834333,25.154039	High	Low
36	-14.735945,24.723264	Low	High
41	-14.716412,24.812838	Low	High
42	-14.772174,24.773974	Low	High
55	-14.541559,24.953973	Low	High
64	-14.817993,24.999905	Low	High
40_1	-14.673717,24.803069	Low	Low
40_2	-14.664325,24.777758	Low	Low
56_2	-14.584204,24.930716	Low	Low
69	-14.814182,25.081806	Low	Low
85	-14.806152,24.742191	Low	Low

References

Chidumayo, E.N. (1987). Species structure in Zambian miombo woodland. Journal of Tropical Ecology, 3, 109-118.

Fanshawe, D.B. (1969). Forest Research Bulletin No. 7 – The Vegetation of Zambia. Lusaka, Zambia: Ministry of Rural Development, Division of Forest Research.

Fanshawe, D.B. (2010). In J.R. Timberlake and M.G. Bingham (Eds.). Occasional Publications in Biodiversity No. 22. Vegetation Descriptions of the Upper Zambezi Districts of Zambia (pp. 87-91). Bulawayo, Zimbabwe: Biodiversity Foundation for Africa.
