# Supplementary material for: Characteristics of the Western Province, Zambia, trial site for evaluation of attractive targeted sugar baits for malaria vector control
Source: Malar J. 2024 May 18;23:153. doi: 10.1186/s12936-024-04985-0 (PMC11102358; doi:10.1186/s12936-024-04985-0)
Supplement: Supplementary file 2 — Supplementary material 2. [file 12936_2024_4985_MOESM2_ESM.pdf]

Vegetation Transect Survey
Kaoma, Nkeyema, Luampa Districts
Western Province, Zambia

PATH/Innovative Vector Control Consortium (IVCC)

Attractive Targeted Sugar Bait (ATSB) Project

February/March 2021

Nicholas Wightman

General Overview

The Innovative Vector Control Consortium (IVCC) is piloting the use of possible methods to reduce mosquito presence around habitation sites through the use of the Attractive Targeted Sugar Bait (ATSB) traps, which ideally will attract feeding mosquitos. However, the flora around habitation sites also provide natural sugar sources for feeding mosquitos from local nectar producing plants. Kaoma, Nkeyema and Luampa Districts of Western Province, Zambia fall within 1100 and 1200 masl with an average rainfall hovering between 800 and 900 mm per year. Chidumayo (1987) designates the flora area from the edge of Mumbwa district to Mongu as western drier miombo, consisting of *Brachystegia spiciformis – Julbernardia paniculata* woodlands with *Burkea africana* as a common canopy co-dominant and *Diplorhynchus condylocarpon* as a common understorey species. The Forest Research Pamphlet on the Vegetation of Mankoya District (present day Kaoma, Nkeyema and Luampa Districts) by D.B Fanshawe was never published at the time in 1964, but has since been published in the larger document, Vegetation Descriptions of the Upper Zambezi Districts of Zambia (Fanshawe, 2010), albeit without the usual woody species list that accompanied his other district vegetation pamphlets. According to Fanshawe, the district (Kaoma, Nkeyema and Luampa) comprises:

- closed forest (dry evergreen as well as riparian forest);
- woodland (Miombo, Kalahari, Chipya and Savanna types);
- dambo grasslands (dry dambos, seepage dambos and sand plains);
- termitaria, where conditions exist for their establishment.

Fanshawe (1969) in The Vegetation of Zambia, describes the dry evergreen forest component in Mankoya (Kaoma, Nkeyema and Luampa) as chiefly *Cryptosepalum* forest with the canopy dominated by *Cryptosepalum exfoliatum* subsp. *pseudotaxus* and *Guibourtia coleosperma* with common understorey woody species *Baphia massaiensis*, *Diplorrhynchus condylocarpon*, *Bauhinia mendoncae*, and *Paropsia brazzeana*. Riparian forest was not observed. The woodland component is a mixture of Miombo and Kalahari with the canopy dominated by *Brachystegia spiciformis*, *Brachystegia longiflora, Julbernardia paniculata, Erythrophleum africanum* and *Burkea africana* and common understorey woody species *Diplorrhynchus condylocarpon, Combretum zeyheri, Terminalia sericea, Hymenocardia acida, Ochna leptostachya* and *Baphia massaiensis*. Chipya and savanna type woodlands are characterised by a more open parkland style plant arrangement with trees spread at greater distances and tall grasses dominating the undergrowth. Due to the higher temperatures of annual fires (the result of the greater grass biomass), tree species are fire tolerant and the herbaceous perennial species will survive the long dry season and annual fires as woody roots, rhizomes or bulbs below ground. Large areas of dambo grasslands border stream and river edges as well as low depressions flanking woodlands and are dominated by grasses and sedges with perennial forbs and suffrutex species growing in lesser numbers. Termitaria are rare due to the high sand content of the soils and usually are only found in the miombo woodland areas where they is enough of a clay content to allow for the construction of their mounds.

In the nearly 60 years since Fanshawe started his work on the vegetation of Mankoya District, much has changed with increased population pressure exerted on the landscape; clearance for agriculture; commercial exploitation of timber tree species and charcoal production. All these factors have, and are still reducing the amount of woody vegetation of the Districts and its associated herbaceous flora.

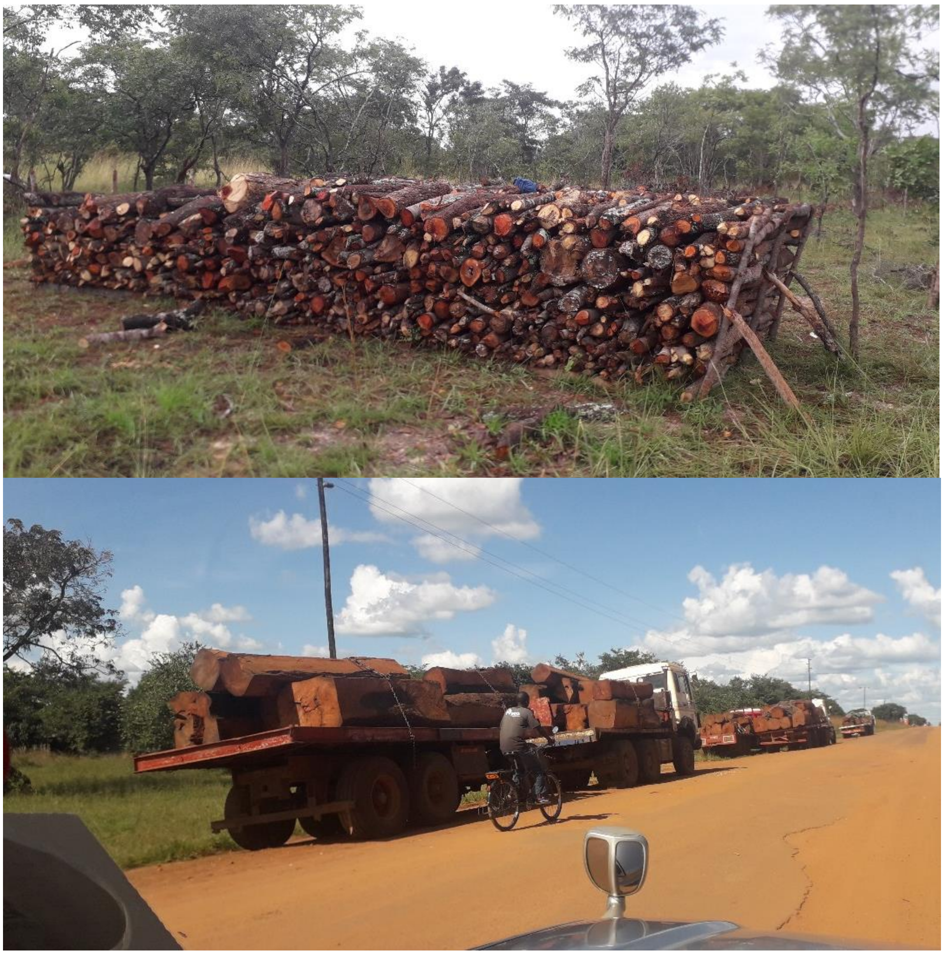

Figure 1. Charcoal production (whether during woodland clearance for agricultural expansion or as a primary activity) and commercial timber harvest are degrading large areas of all three study districts (Photographs: Nicholas Wightman, March 2021).

Methodology

The methodology of the current fieldwork was provided by the IVCC though slightly modified due to possible concerns over conditions of the flora around the possible households within the study clusters. As such, the methodology used during fieldwork in detailed in Appendix I at the end of this document.

Results and Discussion

The structure of the village unit is based on each individual household with the village name usually following the name of the head of the household. Agricultural plots are spread around each household with typical sizes ranging from approximately 1 – 2 hectares in size with maize and cassava occupying the largest areas of production and smaller plots of groundnut, sweet potato, sorghum and millet where they can be accommodated (Figure 2). In most instances, there are some areas left fallow during each growing season and in such areas vegetation may be allowed of grow or may be repeatedly slashed during the growing season (Figure 3). Areas directly surrounding each household structure are usually swept clean with little to no vegetation aside from shade or fruit trees and occasionally ornamentals in pots or bags that are maintained for aesthetic purposes.

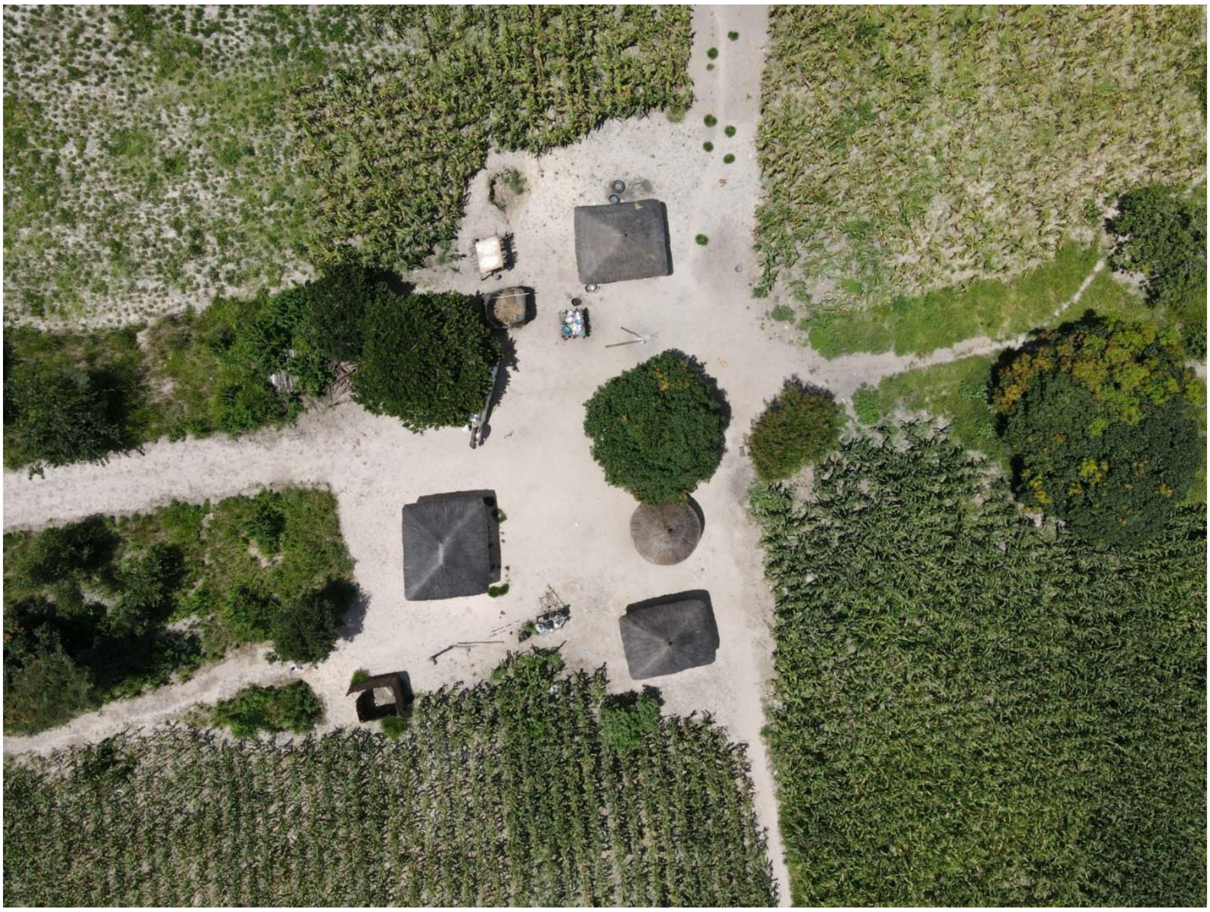

Figure 2. Drone view of one of the households in Cluster 84 showing the extent of agricultural cultivation around the household, the clearance of vegetation directly around each household structure except for shade and fruit trees and sparse areas of fallow land (Photograph: Nicholas Wightman, March 2021).

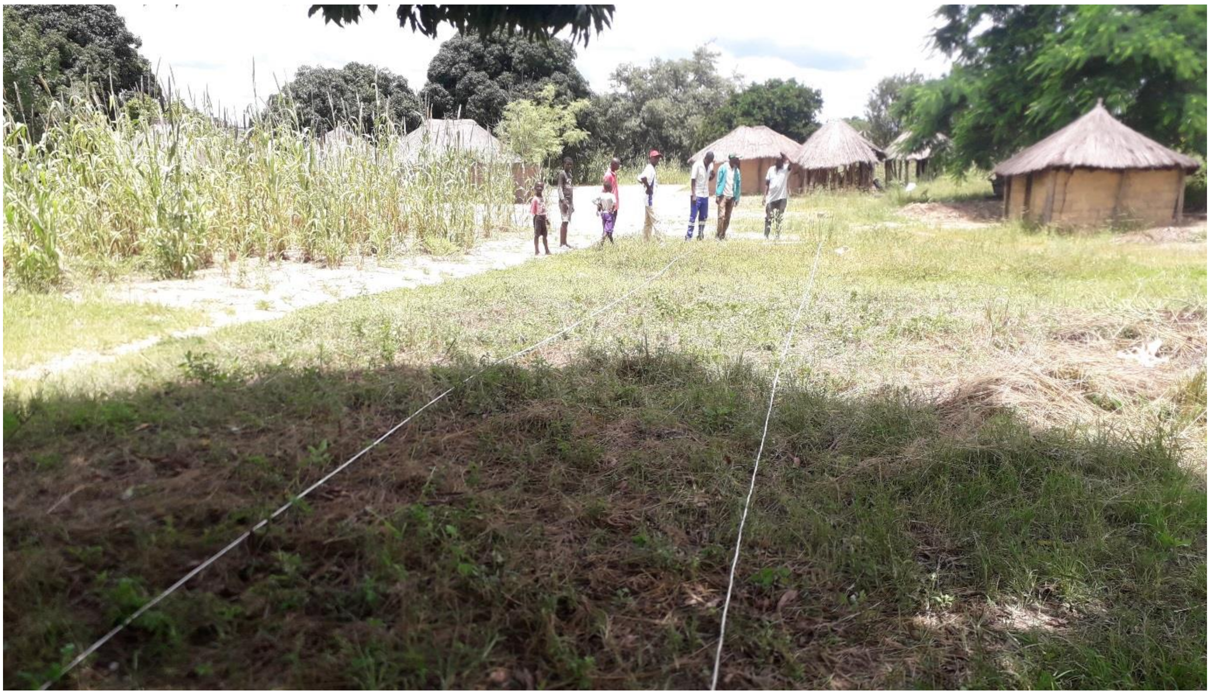

Figure 3. One of the transects where the vegetation is subject to slashing during the rains. Despite the slashing, many annuals are still able to recover or adapt and flower profusely (Photograph: Nicholas Wightman, Feb. 2021).

The results of each transect and household tree data figures are documented in MS Excel files submitted in addition to this report. Each file covers one cluster, labelled appropriately, with two tabs per household indicated by the householders surname with the accompanying designation A, for each transect nearest to the household structures, and B, for each transect set slightly further away but within 100 m of the household structures.

<u>Vegetation breakdown</u>

The vegetation most commonly encountered in transects closest to the household structures were weedy annual plants of both exotic and indigenous origin. Frequently encountered exotic annuals included *Richardia scabra, Bidens pilosa, Acanthospermum hispidulum* and *Ludwigia erecta* while some commonly encountered exotic species included *Euphorbia hirta, Acanthospermum glabratum, Acmella radicans, Xanthium strumarium* and *Portulaca oleracea*. Equally, there were frequently encountered indigenous annuals including *Bidens schimperi, Indigofera nummulariifolia, Vernonia meiostephana, Laggera crispata, Leucas marticiensis* and *Zornia glochidata*. The disturbed nature of the soils directly surrounding the household structures favours the growth and proliferation of these annuals many of which also grow prolifically within the agricultural areas between crop rows. In some transects, the density of some species, chiefly *Richardia scabra, Bidens schimperi, Indigofera nummulariifolia* and *Vernonia meiostephana,* would number several hundred plants per quadrat; in such cases, the plants were small and spindly, producing few flower stalks and seed individually but as a clump, producing more than what several large individual plants would.

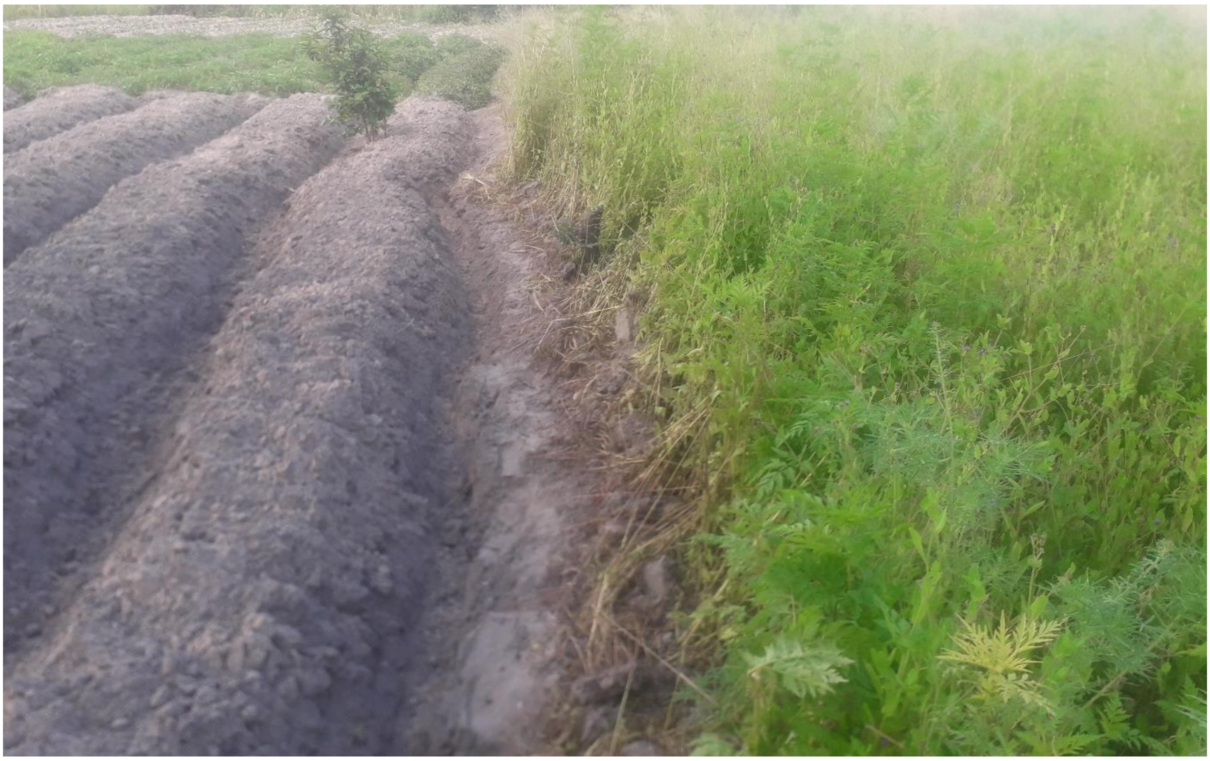

Figure 4. Newly cultivated mounds ready for the planting of sweet potato beside an area of fallow field where the proliferation of *Vernonia meiostephana* and *Bidens schimperi* plants numbered into the hundreds of plants per quadrat (Photograph: Nicholas Wightman, March 2021).

In addition, species of plant used by the householders were regularly encountered including *Hibiscus acetosella, Ceratotheca sesamoides, Sesamum calycinum* and occasionally *Amaranthus hybridus*, the of which are picked and eaten as well as *Sida alba*, a small to medium sized shrub in which the stems are used as a makeshift broom. Occasionally, crop plants were also encountered, usually regrowing from the previous season's crop (i.e. cassava, groundnuts, sweet potato). Larger trees and shrubs were most often absent from transects closest to the household structures and when present, were usually only young seedlings or coppice regrowth from persistent roots.

In contrast, the vegetation in the transects slightly removed from the household structures was usually more diverse with a greater mix of woody, herbaceous perennial and annual species. The areas in particular were usually further removed from agricultural plots on the periphery of the households beside areas of degraded woodlands or fallow land that is unsuitable for agriculture. Several of the common annuals encountered in transects nearer the household structures such as *Indigofera nummulariifolia, Richardia scabra, Bidens schimperi*, *Zornia glochidata* and *Vernonia meiostephana* were observed but usually in much fewer numbers and fewer quadrats per transect. In these less disturbed areas more diversity of annuals were found with species such as *Kohautia caespitose* subsp*. brachyloba, Gutenbergia gossweileri, Vernonia perrottetii, Commelina aspera* var. *aspera* as well as a few species each of *Spermacoce*, *Crotalaria*, *Tephrosia* and *Indigofera*. Shrubs were also much more common with Clerodendron buchneri, Sclerocroton oblongifolius, Paropsia brazzeana, Bauhinia urbiniana, Bauhinia mendoncae and Phyllanthus welwitschianus regularly observed. Fewer herbaceous perennials plants were in flower during the timing of fieldwork activities and positive identifications based on sterile or fruiting material was not always possible to a definitive species level but instead assigned to an affinity of a certain species (i.e. Dolichos sp. aff. linearifolius). Tree species were also much more common but again as saplings or coppice regrowth and rarely as full grown specimens.

In relation to the main objective of flowering plants that could be natural nectar sources for mosquito populations, it is presumed that smaller flowered species in which the corolla tube is more easily accessible by a small or short proboscis would be more frequently visited by feeding mosquitos. In such case, Appendix II provides a list of plants encountered in which the flowers are small enough for feeding mosquitoes with some plants pictured in Figure 5. A good proportion of the plants listed in Appendix II are annual species with many occurring in the sunflower family (Asteraceae), pea family (Fabaceae – Faboideae) and the coffee family (Rubiaceae).

Conclusion

The timing of fieldwork was well timed in the latter half of the rainy season allowing for identification of all the annual plant species and the majority of herbaceous and woody perennial species encountered. Further fieldwork for the identification of plant species in the latter half of the rainy season would be unnecessary unless the scope of work were to be expanded to greater areas farther away from households. Those species identified close to each household are in a cyclical system of cultivation and soil disturbance that allows their regeneration and proliferation. Uncultivated and less disturbed areas provides habitat for greater plant species diversity which was only just touched upon by the current fieldwork. Any future fieldwork, if necessary, might be best concentrated during the early parts of the rainy season, during November or December, as the late dry season and early rainy season perennials start to bloom. However, such plants would most likely not exist close to the households but nearer to each household periphery as well as in the remaining natural woodland and dambo grassland habitats.

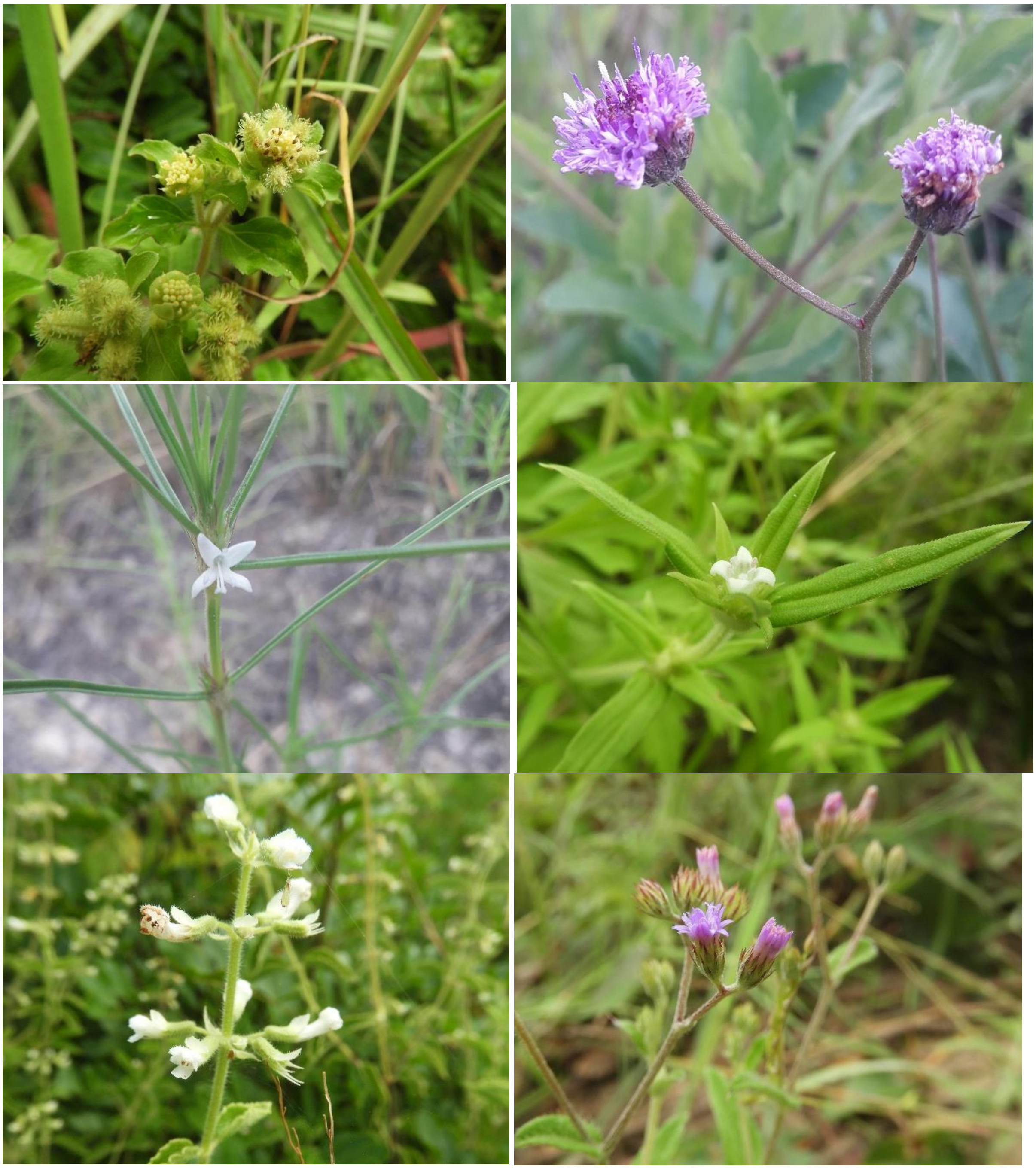

Figure 5. Selection of small flowered annual or perennial herbs that mosquito could possibly be able to feed from. Top row: *Acanthospermum glabratum* (left) and *Vernonia meiostephana* (right); Middle row: *Spermacoce subvulgata* (left) and *Spermacoce senensis* (right); Bottom row: *Ocimum lamiifolium* (left) and *Gutenbergia gossweileri* (Photographs: Nicholas Wightman, Feb./March 2021).

References

Chidumayo, E.N. (1987). Species structure in Zambian miombo woodland. *Journal of Tropical Ecology*, 3, 109-118.

Fanshawe, D.B. (1969). Forest Research Bulletin No. 7 – The Vegetation of Zambia. Lusaka, Zambia: Ministry of Rural Development, Division of Forest Research.

Fanshawe, D.B. (2010). In J.R. Timberlake and M.G. Bingham (Eds.). Occasional Publications in Biodiversity No. 22. *Vegetation Descriptions of the Upper Zambezi Districts of Zambia* (pp. 87-91). Bulawayo, Zimbabwe: Biodiversity Foundation for Africa.

Appendix I

Procedure

a) Estimation of vegetation %cover, frequency, Density and abundance.

1. Visit the 10 ATSB feeding study clusters in Kaoma, Luampa, and Nkeyema Districts, Western Province, Zambia, between February 24 and March 27, 2021.

2. Randomly select 5 households in each cluster and seek an oral consent from household owners and local administrative unit (LAU) to carry out a vegetation survey.

3. Sample two areas within each household near inhabited structures at points **A** and **B** as space within each household allows.

4. Set up a 2m by 20m belt transect over the vegetation at points **A** and **B** consecutively using the 200m nylon string and surveyors tape measure.

5. Identify all the plants enclosed by the belt transect to species level classifying each as either a tree, shrub or herb growth forms. For plants species unable to be identified during fieldwork, collection specimens were made for final determination when back in the office in Lusaka.

6. Count all individual species of **trees** enclosed in the 2m by 20m belt transect and record each species tallies in the data sheet. For **shrubs** and **herbs**, place the gridded quadrat at **0** mark of the belt and sample along the belt transect to the end to obtain **10 replicate** quadrats in each of the sampling sites A and B. Record each species % cover and tallies in the data sheets. Two data sheets will be filled out for each belt transect laid.

7. Calculate the percentage **frequency**, **density**, **cover** and **abundance** of various growth forms (trees, shrubs and herbs) enclosed by the belt transect using the formulae described below. The **frequency** of individual species is the number of times the species occur in the sampling unit or the degree of dispersal of species usually represented as a percentage. **Density** refers to the number of individual species per unit area. **Abundance** is the total number of individual species in all the quadrats in which they occur. **Cover** refers to the proportion of the ground obscured by a species aboveground leaves, stems and flowers.

I. **Abundance**= $\frac{\text{Total no. of individual of the species}}{\text{No. of quadrats in which they occur}}$ X100

II. **Density** = $\frac{\text{Total no. of individual of the species}}{\text{Total no. of quadrats studied}}$ X100

III. **Relative abundance** = $\frac{\text{Total no. of species A}}{\text{Total no. of quadrats in which they occur}}$ x 100

IV. **Relative Density** = $\frac{\text{Density of a given species}}{\text{Total densities of all the species}}$ X100

V. **Frequency**= $\frac{\text{No. of quadrats in which the species occurred}}{\text{Total no. of quadrats studied}}$ X100

8. Estimation of vegetation cover in a gridded quadrat:

i). Lay the 2m by 2m quadrat within the 2m by 20m belt transect and estimate the % cover by dividing down method as follows:

ii). If the vegetation in the quadrat **is consolidated** (not scattered over the quadrat), divide the quadrat into grids so that each grid-square represents 1% cover and if present in all the grids then, 100% cover. Measure cover within the quadrat even if the plant is rooted outside the quadrat **but** do not measure cover outside the quadrat even if the plant is rooted within the quadrat?

iii). If the plants are **scattered** across the quadrat, estimate the cover of each plant and add them together for complete cover of the species in the quadrat. Assume each plant covers 0.5% within the grid. e.g. (0.5%+ 0.5%+0.5%+0.5%) =2.0% cover

iv). If a species cover almost all of the quadrats, estimate the parts of the quadrat without the species and deduct the value from 100%. e.g. if a species fails to cover only 6% of the quadrat, so the specie's cover is about 94%. Enter the cover estimation per quadrat in data sheet in appendix 2.

9. Using a high quality camera take photograph images of the vegetation in the sampled areas for future reference and write down in a note book the compound and cluster number, Date and photograph ID.

b) Estimation of tall vegetation cover using a drone.

NB: Before launching the survey, seek permission from the local aircraft authority and land owners to operate the Unmanned Aerial Vehicle (UAV) /Drone. Test camera resolution at different elevations and ensure you have spare battery in case the survey will take more than a 30 minutes flight.

i) Use a drone (DJI-Mavic-Air- 2- combo) to take still aerial photographs of each of the clusters at 90° from a height of between **50m.** The camera should record the GPS coordinates and elevation of major features; representative plants or vegetation cover to allow for ground-truthing of these points with aerial images.

ii) Make a list of all flowering plant species found around the household which could be sources of sugar for mosquitoes.

iii) Fly the drone over the compound to capture the images of all the tall vegetation keeping visual contact with the aircraft.

iv) **Processing and analysis of image data**: After landing of the Unmanned Aerial Vehicle ((UAV), all recorded images are downloaded to the computer. Select one quality image which has all the representative flowering vegetation around the household for analysis. Download and install the app **imageJ**. Open the software and import the photo to be analyzed from the computer and crop it. Transform the photo to binary image. Go to Image>Process>make binary and once the process is complete, ensure the image is 8-bit format. Choose elliptical selection tool which allows you to draw circles and eclipses. With outline of the image as the guide, draw a circle around the area you want to measure the canopy cover of. Go to analyze>Measurement to compute the canopy area of the

highlighted image. Estimate canopy area of each plant species at a time. Get the total canopy area for each plant species. Repeat this procedure for all the remaining plant species and get the total canopy area of all the plants. Compute the proportion of each plant species by expressing its total canopy area over the canopy area of all the plants around the household. Multiply each plant species proportion by 100 to obtain the percentage canopy cover.

Appendix II

Table 1. Small flowered plants encountered in transects in one or more Clusters in the study area.

Species	Family
Acanthospermum glabratum	Asteraceae
Acanthospermum hispidulum	Asteraceae
Aspilia pluriseta subsp. *pluriseta*	Asteraceae
Bidens pilosa	Asteraceae
Bidens schimperi	Asteraceae
Crassocephalum bauchiense	Asteraceae
Emilia abyssinica var. *abyssinica*	Asteraceae
Eschenbachia stricta	Asteraceae
Erigeron bonariensis	Asteraceae
Gutenbergia gossweileri	Asteraceae
Laggera crispata	Asteraceae
Vernonia meiostephana	Asteraceae
Vernonia perrottetii	Asteraceae
Vernonia stenocephala	Asteraceae
Wahlenbergia cephalodina	Campanulaceae
Commelina africana	Commelinaceae
Commelina aspera var. aspera	Commelinaceae
Commelina benghalensis	Commelinaceae
Commelina subulata	Commelinaceae
Crotalaria anisophylla	Fabaceae – Faboideae
Crotalaria tenuirama	Fabaceae – Faboideae
Desmodium tortuosum	Fabaceae – Faboideae
Grona cordifolia	Fabaceae – Faboideae
Indigofera charlieriana	Fabaceae – Faboideae
Indigofera dissitiflora var. *major*	Fabaceae – Faboideae
Indigofera monantha	Fabaceae – Faboideae
Indigofera nummulariifolia	Fabaceae – Faboideae
Indigofera polysphaera	Fabaceae – Faboideae
Tephrosia micrantha	Fabaceae – Faboideae
Tephrosia pumila var. *pumila*	Fabaceae – Faboideae
Tephrosia purpurea subsp. *leptostachya* var. *leptostachya*	Fabaceae – Faboideae
Tephrosia purpurea subsp. *leptostachya* var. *pubescens*	Fabaceae – Faboideae
Tephrosia reptans	Fabaceae – Faboideae
Tephrosia rhodesica var. *rhodesica*	Fabaceae – Faboideae
Zornia glochidata	Fabaceae – Faboideae
Ocimum americanum	Lamiaceae
Ocimum lamiifolium	Lamiaceae
Plectranthus bojeri	Lamiaceae
Sida alba	Malvaceae – Malvoideae
Triumfetta rhomboidea	Malvaceae – Malvoideae
Triumfetta setulosa	Malvaceae – Malvoideae
Ludwigia erecta	Onagraceae
Alectra sessiliflora	Orobanchaceae
Buchnera hispida	Orobanchaceae

Buchnera longispicata	Orobanchaceae
Striga asiatica	Orobanchaceae
Biophytum umbraculum	Oxalidaceae
Polygala albescens	Polygalaceae
Polygala baumii	Polygalaceae
Polygala welwitschii subsp. pygmaea	Polygalaceae
Polygala westii	Polygalaceae
Kohautia caespitose subsp. *brachyloba*	Rubiaceae
Kohautia subverticillata subsp. *subverticillata*	Rubiaceae
Multidentia concrescens	Rubiaceae
Psychotria pumila var. *pumila*	Rubiaceae
Richardia scabra	Rubiaceae
Spermacoce huillensis	Rubiaceae
Spermacoce pusilla	Rubiaceae
Spermacoce senensis	Rubiaceae
Spermacoce subvulgata	Rubiaceae
Vangueria cistifolia var. latifolia	Rubiaceae
Lantana camara	Verbenaceae
Ampelocissus obtusata var. *kirkiana*	Vitaceae
Cyphostemma crotalarioides	Vitaceae
Cyphostemma rhodesiae	Vitaceae
